# Efficacy and safety of gout flare prophylaxis and therapy use in people with chronic kidney disease: a Gout, Hyperuricemia and Crystal-Associated Disease Network (G-CAN)-initiated literature review

**DOI:** 10.1186/s13075-021-02416-y

**Published:** 2021-04-28

**Authors:** Huai Leng Pisaniello, Mark C. Fisher, Hamish Farquhar, Ana Beatriz Vargas-Santos, Catherine L. Hill, Lisa K. Stamp, Angelo L. Gaffo

**Affiliations:** 1 Discipline of Medicine, Faculty of Health and Medical Sciences, the University of Adelaide, Adelaide, South Australia Australia; 2 Harvard Medical School and Massachusetts General Hospital, Boston, MA USA; 3 Prima CARE, Fall River, MA USA; 4 Department of Medicine, University of Otago, Christchurch, New Zealand; 5 Department of Internal Medicine, Rio de Janeiro State University, Rio de Janeiro, Brazil; 6 Rheumatology Unit, The Queen Elizabeth Hospital, Woodville South, South Australia Australia; 7 Division of Rheumatology and Clinical Immunology, University of Alabama, 1720 2nd Avenue South, Birmingham, AL 35294 USA; 8 Birmingham VA Medical Center, Birmingham, USA

**Keywords:** Gout, Gout flare, Colchicine, Corticosteroids, Non-steroidal anti-inflammatory, Interleukin 1 inhibitors, Treatment

## Abstract

Gout flare prophylaxis and therapy use in people with underlying chronic kidney disease (CKD) is challenging, given limited treatment options and risk of worsening renal function with inappropriate treatment dosing. This literature review aimed to describe the current literature on the efficacy and safety of gout flare prophylaxis and therapy use in people with CKD stages 3–5. A literature search via PubMed, the Cochrane Library, and EMBASE was performed from 1 January 1959 to 31 January 2018. Inclusion criteria were studies with people with gout and renal impairment (i.e. estimated glomerular filtration rate (eGFR) or creatinine clearance (CrCl) < 60 ml/min/1.73 m^2^), and with exposure to colchicine, interleukin-1 inhibitors, non-steroidal anti-inflammatory drugs (NSAIDs), and glucocorticoids. All study designs were included. A total of 33 studies with efficacy and/or safety analysis stratified by renal function were reviewed—colchicine (*n* = 20), anakinra (*n* = 7), canakinumab (*n* = 1), NSAIDs (*n* = 3), and glucocorticoids (*n* = 2). A total of 58 studies reported these primary outcomes without renal function stratification—colchicine (*n* = 29), anakinra (*n* = 10), canakinumab (*n* = 6), rilonacept (*n* = 2), NSAIDs (*n* = 1), and glucocorticoids (*n* = 10). Most clinical trials excluded study participants with severe CKD (i.e. eGFR or CrCl of < 30 mL/min/1.73 m^2^). Information on the efficacy and safety outcomes of gout flare prophylaxis and therapy use stratified by renal function is lacking. Clinical trial results cannot be extrapolated for those with advanced CKD. Where possible, current and future gout flare studies should include patients with CKD and with study outcomes reported based on renal function and using standardised gout flare definition.

**Supplementary Information:**

The online version contains supplementary material available at 10.1186/s13075-021-02416-y.

## Background

Gout, a highly prevalent inflammatory arthritis worldwide, is often linked with renal impairment, among all other comorbidities clustered within the term ‘metabolic syndrome’ [1, 2]. For instance, in a nationwide US representative study, 19.9% of adults with gout had CKD ≥ stage 3 (i.e. estimated glomerular filtration rate (eGFR) of < 60 mL/min/1.73 m^2^) compared with 5.2% of adults without gout [3]. Gout is also highly prevalent in individuals with pre-existing CKD. In an age-standardised gout prevalence study in the USA, nearly one-fourth of adults with CKD ≥ stage 3 reported having gout in comparison with 2.9% individuals with normal renal function [4]. The degree of renal impairment, especially in advanced CKD, invariably plays a major role in the treatment decision-making when managing gout.

Gout flares, when inadequately treated, can have a profound impact on physical functioning and quality of life [5]. According to the 2020 American College of Rheumatology (ACR) guideline, colchicine, non-steroidal anti-inflammatory drugs (NSAIDs), and parenteral/oral glucocorticoids are recommended as the preferred first-line treatment options for managing gout flares [6, 7]. These anti-inflammatory treatment options are also recommended as short-term prophylaxis for when commencing urate-lowering therapy (ULT) [6, 7]. However, the use of gout flare prophylaxis and therapy in people with CKD is not always straightforward. In the context of minimal or absence of residual renal function, treatment options for gout flare are limited, with potential risks of further renal impairment. Renally adjusted dosing is often required in people with CKD, although there is no specific evidence-based guidance in monitoring the efficacy and safety of the treatment used. Therefore, clinicians often remain judicious when facing this common clinical conundrum in the management of gout flares. It is reassuring that, based on a recent systematic review, colchicine use is relatively safe in all possible clinical indications, with diarrhoea and gastrointestinal symptoms being the most commonly reported adverse events [8]. However, no conclusion could be precisely drawn from this review on the safety profile of colchicine use in people with CKD [8]. Overall, for all anti-inflammatory drugs used for gout flare, lack of consensus on the appropriate dosing and treatment monitoring for this high-risk comorbid population remains. The extent of this deficiency in the literature in terms of the efficacy and safety data for gout flare prophylaxis and therapy is unknown.

Accordingly, in parallel with the mission of the Gout, Hyperuricemia and Crystal-Associated Disease Network (G-CAN), this G-CAN-initiated literature review aims to identify all available literature on gout flare prophylaxis and therapy use in people with CKD stages 3–5. In detail, we aim to explore the best available evidence that we currently have on the efficacy and safety of gout flare prophylaxis and therapy in this high-risk comorbid population, alongside the identification of important key areas for future research on this issue.

## Methods

This literature review was conducted in accordance to the Preferred Reporting Items for Systematic Reviews and Meta-Analyses (PRISMA) guidelines.

### Literature search strategy

A literature search in PubMed, The Cochrane Library, and EMBASE was conducted from 1 January 1959 to 27 June 2017. A subsequent search from 28 June 2017 to 31 January 2018 was updated to capture any additional studies published during the review process. We included all available gout flare prophylaxis and therapy use in clinical trials and real-world practice, which were colchicine, NSAIDs, glucocorticoids, and interleukin-1 (IL-1) inhibitors such as anakinra, canakinumab, and rilonacept.

In detail, literature search combining gout with either gout flare prophylaxis/therapy and CKD was performed separately to the literature search combining either gout flare prophylaxis/therapy and renal replacement therapy (i.e. haemodialysis and peritoneal dialysis). These two literature searches were subsequently merged prior to the screening phase. This search strategy was applied to all gout flare prophylaxis/therapy, except for glucocorticoids. The initial search attempt in crossing the glucocorticoid-related search terms with renal-related terms led to an excessive number of irrelevant search results. Therefore, for glucocorticoid-related literature search, the search term was only crossed against gout terms, and not with any renal-related terms. The search strategies for each database were outlined in Supplementary Table 1.

### Eligibility criteria and study selection

We included studies which fulfil the following criteria: people diagnosed with gout, with CKD ≥ stage 3 (i.e. eGFR or creatinine clearance (CrCl) of < 60 mL/min/1.73 m^2^), and with exposure to the gout flare prophylaxis/therapy of interest. Only studies published in English were included. Studies in the form of case reports or case series as well as abstracts from the ACR and the European League Against Rheumatism (EULAR) were included for screening.

We excluded studies if the primary study population had a diagnosis other than gout, studies with inadequate or absence of information on the renal function (i.e. absence of CKD stage or eGFR/CrCl measure) and/or on the study drug of interest, and studies that included people with normal renal function or experiencing acute renal failure. In addition, studies published in the form of letters, editorials, opinions, review articles, and studies with animal-, basic science- or laboratory-based focus were excluded.

Study title and abstract screening for eligible studies was independently performed by two reviewers (HLP and CLH for colchicine and IL-1 inhibitors; MCF and AG for NSAIDs and glucocorticoids). Full-text screening for eligible studies for data extraction was independently performed by two reviewers in similar arrangement. Any discrepancy identified during the screening phase was discussed to reach consensus.

### Data extraction

Relevant data for eligible studies were extracted independently by two reviewers (HLP and CLH for colchicine and IL-1 inhibitors; MCF and AG for NSAIDs and glucocorticoids). The extracted data included the primary author, year of study, trial name (where applicable), study design, and sample size. The extracted outcome data included the efficacy of the drug of interest (defined as the clinical resolution of gout flare or absence of gout flare during concomitant ULT use) and/or the safety profile of the drug of interest (defined as adverse events observed in the presence of active use of gout flare prophylaxis/therapy). Where applicable, we extracted studies reporting these outcome data as stratified by renal function. Discrepancies among the reviewers during this data extraction phase were minimal and were resolved by discussion.

### Analyses

The eligible studies were analysed in terms of their overall study characteristics, sample size, drug indication (i.e. either as gout flare prophylaxis or as gout flare treatment), and dosages, and the efficacy and safety outcomes for the study drug of interest and the corresponding renal function stratification, where applicable. We were not able to summarise these studies quantitatively due to the heterogeneity nature of the studies included.

## Results

An overview for all included studies was outlined in Table 1. Herein, results for each gout flare prophylaxis/therapy with efficacy and safety outcome data stratified by renal function were presented in the main text and were summarised in Table 2. Specifically, the details for drug indication and drug dosage were reported in Table 2. For studies with outcome data reported without any renal function stratification, these were summarised separately in the Supplementary Materials.

**Table 1:** An overview of efficacy and safety outcome reporting of gout flare prophylaxis and therapy use (with and without renal function stratification)

First Author (Year) (Trial Name)	Study Design	Number of participants by eGFR/CrCl at baseline (mL/min/1.73m^2^)				Total, n	Primary outcome data reporting with stratified renal function = Yes without stratified renal function = No	
		≥90	60-90	30-60	< 30		Efficacy	Safety
**COLCHICINE**								
*AHERN 1987* [9]	*RCT (single- centre)*	*22*				*22*	*No*	*No*
*BORSTAD 2004* [10]	*RCT (single-centre)*	*14*				*14*	*No*	*No*
*WORTMANN 2010 (FACT, APEX, CONFIRMS)* [11]	*Three RCTs (post-hoc analyses)*	*371(F); 541(A); 786(C)*	*295(F); 154(A); 402(C)*	94(F); 154(A); 402(C)		*760(F); 1072(A); 2269(C)*	*No*	*No*
*PASCART 2016 (GOSPEL 2)* [12]	*Cross-sectional study (post-hoc analysis)*	*158*	*59*	*45*	2	*264*	*No*	*No*
*SOLAK 2014* [13]	*Case-control*				*1*	*1*	*No*	*No*
*HUNG 2005* [14]	*Retrospective observational (single-centre)*	*29 (concomitant arm) vs 8 (sequential arm)*				*37*	*No*	*No*
*KWON 2017* [15]	*Retrospective observational (single-centre)*	*36/188 (colchicine with statin arm) vs 41/486 (colchicine without statin arm)*				*77*	*No*	*No*
*AKAR 2001* [16]	*Case report*				*1*	*1*	*No*	*No*
AKDAG 2006 [17]	Case report				1	1	No	Yes
ALAYLI 2005 [18]	Case report			1		1	Yes	Yes
*ALTIPARMAK 2002* [19]	*Case series*				*1*	*1*	*No*	*No*
ALTMAN 2007 [20]	Case report			1		1	No	Yes
*BAKER 2004* [21]	*Case report*			*1*		*1*	*No*	*No*
BONNEL 2002 [22]	Case series				1	1	No	Yes
*BOOMERSHINE 2002* [23]	*Case report*				*1*	*1*	*No*	*No*
BOUQUIÉ 2011 [24]	Case report			1		1	No	Yes
*CHENG 2005* [25]	*Case series*				*1*	*1*	*No*	*No*
*CHOI 1999* [26]	*Case report*				*1*	*1*	*No*	*No*
*DIXON 2001* [27]	*Case report*			*1*		*1*	*No*	*No*
ELEFTHERIOU 2008 [28]	Case report				1	1	No	Yes
GARROUSTE 2012 [29]	Case report				1	1	No	Yes
*HSU 2002* [30]	*Case report*				*1*	*1*	*No*	*No*
HUH 2013 [31]	Case report			1		1	No	Yes
JUSTINIANO 2007 [32]	Case report			1		1	No	Yes
KUBLER 2000 [33]	Case report				1	1	Yes	Yes
*KUNCL 1987* [34]	*Case series*	*12*				*12*	*No*	*No*
LAI 2006 [35]	Case report				1	1	No	Yes
LEE 1997 [36]	Case report			1		1	No	Yes
LY 2007 [37]	Audit (single centre)		22			22	No	Yes
*MARCINIAK 2016* [38]	*Case report*			*1*		*1*	*No*	*No*
MEDANI 2016 [39]	Case series			1	1	2	No	Yes
*MONTSENY 1996* [40]	*Case series*				*4*	*4*	*No*	*No*
*MORRIS 2003* [41]	*Case series*				*1*	*1*	*No*	*No*
*MULLINS 2011* [42]	*Audit (single centre)*	*4*	*7*	*16*	*10*	*37*	*No*	*No*
*NASHEL 1982* [43]	*Case series*				2	2	*No*	*No*
NEUSS 1986 [44]	Case report				1	1	Yes	Yes
*ORTEL 1974* [45]	*Case report*			*1*		*1*	*No*	*No*
PATEL 2016 [46]	Case report			1		1	No	Yes
*PETERSEL 2007* [47]	*Audit (single centre)*	*38*				*38*	*No*	*No*
*RANA 1997* [48]	*Case series*			*3*		*3*	*No*	*No*
RIEGER 1990 [49]	Case report				1	1	No	Yes
*RUTKOVE 1996* [50]	*Case series*				*4*	*4*	*No*	*No*
*SU 2015* [51]	*Case report*				*1*	*1*	*No*	*No*
*VAN DER VALDEN 2008* [52]	*Case report*				*1*	*1*	*No*	*No*
WILBUR 2004 [53]	Case series			1	1	2	Yes	Yes
*WRIGHT 2017* [54]	*Audit*	*128*				*128*	*No*	*No*
YOON 2001 [55]	Case report			1		1	Yes	Yes
*YU 2018* [56]	*Audit*	*9*				*9*	*No*	*No*
ZAGLER 2009 [57]	Case report			1		1	No	Yes
**IL-1 INHIBITORS**								
*SCHLESINGER 2011* [58]	*Phase 2 RCT – an extension of a phase 2 RCT by So et al., 2010* *(multi-centre)*	*95*				95	*No*	*No*
*SCHLESINGER 2012* *(β-RELIEVED & β-RELIEVED-II)* [59]	*Two phase 3 RCTs, followed by extension studies for both trials (multi-centre)*		*123*	59		*182*	*No*	*No*
*SO 2007* [60]	*Pilot, open-labelled study (single-centre)*	*2*	*5*	2	*1*	*10*	*No*	*No*
*SO 2010* [61]	*Phase 2 RCT (multi-centre)*	*95*				*95*	*No*	*No*
*SUNKUREDDI 2011* [62]	*Post-hoc analyses of the RCTs (β-RELIEVED & β-RELIEVED-II)* ACR abstract		*188*			*188*	*No*	*No*
*SUNKUREDDI 2013* [63]	*Post-hoc analyses of the RCTs (β-RELIEVED & β-RELIEVED-II)* *EULAR abstract*		*65*			*65*	*No*	*No*
*SUNKUREDDI 2014* [64]	*Post-hoc analysis of an RCT (multi-centre)* *ACR abstract*			*24*		*24*	*No*	*No*
*TERKELTAUB 2009* [65]	*Crossover trial (multi-centre)*	*2*				*2*	*No*	*No*
*TERKELTAUB 2012* [66]	*Post-hoc analyses of RCTs (PRE-SURGE 1, PRE-SURGE 2 and RE-SURGE)* *ACR abstract*		*624*	*103*		*727*	*No*	*No*
ADLER 2017 [67]	Case report				1	1	Yes	No
AOUBA 2015 [68]	Case series (single-centre)	1	1	1		3	Yes	Yes
BARTOV 2013 [69]	Case report				1	1	Yes	Yes
*CHEN 2010* [70]	*Case series (single-centre)*		*2*	*5*	*3*	*10*	*No*	*No*
DIREZ 2012 [71]	Case report				1	1	Yes	Yes
*DONMEZ 2014* [72]	*Case report*			*1*		*1*	*No*	*No*
*FUNCK-BRENTANO 2011* [73]	*Case report*				*1*	*1*	*No*	*No*
*GHOSH 2013* [74]	*Case series (single-centre)*			*5*		*5*	*No*	*No*
*GRATTON 2009* [75]	*Case report*				*1*	*1*	*No*	*No*
LOUSTAU 2018 [76]	Case series (multi-centre)			6	25	31	Yes	Yes
MAROTTO 2018 [77]	Case report			1		1	Yes	Yes
*MCGONAGLE 2007* [78]	*Case report*			*1*		*1*	*No*	*No*
*OTTAVIANI 2013* [79]	*Case series (multi-centre)*			*40*		*40*	*No*	*No*
*PALMA 2016* [80]	*Case series (single-centre)* *ACR abstract*			*18*		*18*	*No*	*No*
PEREZ-RUIZ 2013 [81]	Case series (single-centre) EULAR abstract	2		6		8	Yes	Yes
*SINGH 2009* [82]	*Case report*			*1*		*1*	*No*	*No*
TRAN 2011 [83]	Case series			1		1	Yes	No
**NON-STEROIDAL ANTI-INFLAMMATORY DRUGS**								
KAHL 1989 [84]	Case series			1	1	2	Yes	Yes
*MIKHNEVICH 2013* [85]	*Case series*	*82*		*15*		*97*	*No*	*No*
SCHLONDORFF 1993 [86]	Case report		1			1	Yes	Yes
ZAGLER 2009 [57]	Case report			1		1	Yes	Yes
**GLUCOCORTICOIDS**								
*SUNKUREDDI 2014* [64]	*Post-hoc analysis of an RCT (multi-centre)* *ACR abstract*			*24*		*24*	*No*	*No*
*BAJAJ 2004* [87]	*Case series (single-centre)*	*4*		*2*	*4*	*10*	*No*	*No*
*FARGETTI 2012* [88]	*Case report*			*1*		*1*	*No*	*No*
*HAUSCH 1991* [89]	*Case report*			*1*		*1*	*No*	*No*
*HILL 2008* [90]	*Case report*				*1*	*1*	*No*	*No*
*KARIMZADEH 2009* [91]	*Case report*			*1*		*1*	*No*	*No*
*MAEKAWA 2014* [92]	*Case report*				*1*	*1*	*No*	*No*
*RICHETTE 2006* [93]	*Case report*				*1*	*1*	*No*	*No*
*SARMENTO 2009* [94]	*Case report*			*1*		*1*	*No*	*No*
TAUSCHE 2011 [95]	Case report			1		1	Yes	Yes
*UDAYAKUMAR 2010* [96]	*Case report*			*1*		*1*	*No*	*No*
ZAGLER 2009 [57]	Case report			1		1	Yes	Yes

ACR: American College of Rheumatology; APEX: Allopurinol- and Placebo-Controlled, Efficacy Study of Febuxostat; CONFIRMS: A Phase 3, Randomised, Multicenter, Double-Blind, Allopurinol-Controlled Study Assessing the Efficacy and Safety of Oral Febuxostat in Subjects With Gout; CrCl: creatinine clearance; eGFR: estimated glomerular filtration rate; EULAR: European League Against Rheumatism; FACT: Febuxostat Versus Allopurinol Control Trial in Subjects With Gout; GOSPEL 2: subgroup analysis of GOSPEL (*goutte et observation des stratégies de prise en charge en médecine ambulatoire) survey; IL-1: interleukin-1; PRE-SURGE 1: Preventative Study Against Urate-Lowering Drug-Induced Gout Exacerbations 1; PRE-SURGE 2: Preventative Study Against Urate-Lowering Drug-Induced Gout Exacerbations;* RCT: randomised controlled trial; RE-SURGE: Review of Safety Using Rilonacept in Preventing Gout Exacerbations; β-RELIEVED & β-RELIEVED-II: two phase three randomised studies (response in acute flare and in prevention of episodes of re-flare in gout)

*Rows set in italics include studies described in the supplementary materials*

**Table 2:** Efficacy outcome reporting of gout flare prophylaxis and therapy use with renal function stratification

First Author (Year) (Trial Name)	Study Design	Renal Function Exclusion Criteria or Baseline Renal Function – eGFR/CrCl (mL/min/1.73m^2^) or Serum Creatinine Level	Clinical Indication for Gout Flare	Actual/Mean Gout Flare Prophylaxis and Therapy Dose	Number of Participants by eGFR/CrCl at Baseline (mL/min/1.73m^2^)				Total, n	Efficacy Data with Renal Function Stratification
					≥90	60–90	30–60	< 30		
COLCHICINE										
AKDAG 2006 [17]	Case report	eGFR of 22 (serum creatinine of 3.1 mg/dL)	Gout flare prophylaxis	0.5 mg PO twice daily (for at least 15 years)				1	1	Efficacy data not available. Renal function deteriorated (serum creatinine peaked at 7.6 mg/dL) during an episode of pneumonia requiring antibiotics (clarithromycin and cefepime).
ALAYLI 2005 [18]	Case report	eGFR of 44 (serum creatinine of 1.3 mg/dL)	Gout flare treatment	1.5 mg PO daily (for few days)			1		1	Gout flare resolution was achieved without worsening renal function (serum creatinine of 1.1 mg/dL – baseline of 1.3 mg/dL)
ALTMAN 2007 [20]	Case report	eGFR of 34 (serum creatinine of 2 mg/dL)	Gout flare treatment	1.5 mg PO daily (for at least 1 week)			1		1	Efficacy data not available. Renal function deteriorated (serum creatinine peaked at 2.56 mg/dL).
BONNEL 2002 [22]	Case series	eGFR of 29 (serum creatinine of 2.3 mg/dL)	Gout flare treatment	2 mg IV loading dose, followed by 0.5 mg IV every 2 h until diarrhoea developed (total dose of 5.5 mg in 5 h)				1	1	Efficacy data not available. Renal function deteriorated rapidly (serum creatinine peaked at 4.9 mg/dL).
BOUQUIÉ 2011 [24]	Case report	eGFR of 32 (serum creatinine of 216 μmol/l)	Gout flare treatment	1 mg PO three times daily on day 1, 1 mg twice daily on day 2 and 3, and 1 mg once daily for 3 days			1		1	Efficacy data not available (colchicine was self-ceased after day 6). Renal function deteriorated on day 8 (serum creatinine peaked at 370 μmol/l).
ELEFTHERIOU 2008 [28]	Case report	ESRD with eGFR of 6–8	Gout flare treatment	1 mg PO daily (for at least 6 days)				1	1	Efficacy data not available. Renal function was stable.
GARROUSTE 2012 [29]	Case report	Renal transplant with eGFR of 41 (serum creatinine of 160 μmol/l)	Gout flare treatment	3 mg PO daily for 7 days				1	1	Efficacy data not available. Renal function deteriorated (serum creatinine peaked at 512 μmol/l), but gradually recovered on day 34 (serum creatinine of 188 μmol/l).
HUH 2013 [31]	Case report	Renal transplant with eGFR of 34 (serum creatinine of 1.65 mg/dL)	Gout flare treatment	0.3 mg PO twice daily (for at least 3 weeks)			1		1	Efficacy data not available. Renal function deteriorated but returned to baseline 2 weeks after colchicine cessation.
JUSTINIANO 2007 [32]	Case report	eGFR of 57 (serum creatinine of 1.6 mg/dL)	Gout flare treatment	0.6 mg PO twice daily (for at least 2 weeks)			1		1	Efficacy data not available. Renal function deteriorated (serum creatinine peaked at 1.7 mg/dL).
KUBLER 2000 [33]	Case report	eGFR of 26 (serum creatinine of 160 μmol/l)	Gout flare treatment	0.5 mg PO three times daily (for at least 12 days)				1	1	Gout flare resolution was achieved within 48 h, but ARF developed (serum creatinine peaked at 450 μmol/l) in the context of ongoing colchicine use (same dose and frequency).
LAI 2006 [35]	Case report	eGFR of 9 (serum creatinine of 565.8 μmol/l)	Gout flare treatment	0.5 mg PO three times daily for 3 days on a monthly basis (for at least 1 year); recent gout flare treatment with 0.5 mg PO twice daily (for at least 2 weeks)				1	1	Efficacy data not available. Renal function deteriorated (serum creatinine peaked at 680.7 μmol/l).
LEE 1997 [36]	Case report	Renal transplant with eGFR of 49 (serum creatinine of 1.6 mg/dL)	Gout flare treatment	0.5 mg PO twice daily for 3 days			1		1	Efficacy data not available. Renal function deteriorated (serum creatinine peaked at 2.7 mg/dL) but returned to baseline upon colchicine cessation.
LY 2007 [37]	Audit (single centre)	Patients with CKD (defined as serum creatinine of ≥0.17 mmol/L or CrCl of < 0.83)	Gout flare treatment	2.5 mg PO over 24 h or less		22			22	Efficacy data not available.
MEDANI 2016 [39]	Case series	CKD Stage 3b-4 (serum creatinine of 300 μmol/l in patient 1 and 200 μmol/l in patient 2)	Gout flare treatment	Patient 1: 0.5 mg PO three times daily for 6 weeks Patient 2: 0.5 mg PO once daily for at least 6 months			1	1	2	Efficacy data not available. Renal function deteriorated for both patients (serum creatinine peaked at 526 μmol/l in patient 1 and 255 μmol/l in patient 2).
NEUSS 1986 [44]	Case report	eGFR of 23 (serum creatinine of 2.3 mg/dL)	Gout flare prophylaxis	0.6 mg PO twice daily (long term)				1	1	No gout flare during therapy, but with multi-organ failure, including AKI (serum creatinine peaked at 3 mg/dL).
PATEL 2016 [46]	Case report	eGFR of 32 (serum creatinine of 1.87 mg/dL)	Gout flare prophylaxis	0.6 mg PO once daily (for > 5 years)			1		1	Efficacy data not available. Renal function deteriorated (serum creatinine peaked at 2.5 mg/dL) but returned to baseline upon colchicine cessation.
RIEGER 1990 [49]	Case report	ESRD with eGFR of < 30 on haemodialysis and underlying renal transplant	Gout flare prophylaxis	0.6 mg PO twice daily				1	1	Efficacy data not available.
WILBUR 2004 [53]	Case series	Patient 1: ESRD on peritoneal dialysis Patient 2: CKD Stage 3	Gout flare treatment	0.6 mg PO (variable frequency)			1	1	2	Patient 1: ongoing treatment due to recurrent flare (despite 0.6 mg PO three times daily for the first 3 days). Renal function worsened. Patient 2: ongoing treatment due to recurrent gout flare for at least 2 weeks (dosing was increased from 0.6 mg PO once daily to three times daily). Renal function was stable.
YOON 2001 [55]	Case report	eGFR of 39 (serum creatinine of 134 μmol/l)	Gout flare treatment	0.5 mg PO three times daily (total dose of 4.5 mg)			1		1	Gout flare persisted. Renal function deteriorated (CrCl peaked at 30 mL/min/1.73m^2^).
ZAGLER 2009 [57]	Case report	eGFR of 34	Gout flare treatment	1 mg PO once off dosing			1		1	Efficacy data not available. However, renal function deteriorated (serum creatinine increased from 2.4 to 5.2 mg/dL).
IL-1 INHIBITORS										
ADLER 2007 [67]	Case report	eGFR of 27–30	Gout flare treatment	Anakinra 100 mg/d for 3 days				1	1	Complete clinical remission with stable renal function.
AOUBA 2015 [68]	Case series (single-centre)	eGFR of 20–40	Gout flare treatment	Anakinra 100 mg/d (up to 6 days)	1	1	1		3	2 patients achieved complete clinical remission after 5 days of treatment. 1 patient who received initial 3-day treatment required a second course (5-day period) due to recurrent gout flare. Renal function remained stable.
BARTOV 2013 [69]	Case report	eGFR of < 20	Gout flare treatment	Anakinra 100 mg every other day for 1 week				1	1	Complete clinical remission with no worsening renal function.
DIREZ 2012 [71]	Case report	CrCl of 14 mL/min/1.73m^2^	Gout flare treatment	Anakinra 100 mg/d for 5 days, followed by re-dosing of alternate-daily 100 mg dosing for 2 months (commenced 1 week after the first therapy)				1	1	Partial responder for the first course of treatment. During the second treatment, renal function deteriorated after 2 months (CrCl declined to 6 mL/min/1.73m^2^).
LOUSTAU 2018 [76]	Case series (multi-centre)	CKD stage 4–5 (mean eGFR 22 ± 6.6) and history of renal transplant (mean eGFR 41 ± 22.8)	Gout flare treatment	Anakinra 100 mg/d (except in 5 patients who had 100 mg every 48–72 h) for less than a week; in 10 patients, dose was maintained for > 15 days (up to 14 months) with progressive dose spacing due to frequent gout flare			6	25	31	Pooled efficacy outcome: complete clinical remission with a mean of 46 days (range 4–90 days). Decrease in pain VAS from 69.6 ± 13.4 mm to 10.4 ± 15.3 mm and CRP level from 160 ± 133 mg/mL to 11 ± 11 mg/mL. No significant change in eGFR (26.3 pre-treatment vs 26.9 post-treatment).
MAROTTO 2018 [77]	Case report	CKD stage 3 (CrCl of 56.47 mL/min/1.73m^2^)	Gout flare treatment	Canakinumab 100 mg single dose			1		1	Rapid clinical response 12 h after canakinumab administration. No worsening renal function.
PEREZ-RUIZ 2013 [81]	Case series (single centre) EULAR abstract	CKD stage 3–4	Gout flare treatment	Anakinra 100 mg/d (varying duration)	2		6		8	Pooled efficacy outcome: reduction in hsCRP level from 5.7 ± 7.3 to 0.56 ± 1.07 at 6 months post-treatment. Renal function remained stable (baseline CrCl 68 ± 28 vs 74 ± 43 at 6th month).
TRAN 2011 [83]	Case series	eGFR of 57 (serum creatinine of 118 μmol/l)	Gout flare treatment	Anakinra 100 mg/d			1		1	Clinical remission was achieved with CRP reduction. Renal function remained stable.
NON-STEROIDAL ANTI-INFLAMMATORY DRUGS										
KAHL 1989 [84]	Case series	Patient 1: eGFR of 32 (serum creatinine of 2.4 mg/dL) Patient 2: eGFR of 21 (serum creatinine of 3.3)	Gout flare treatment	Sulindac 200 mg twice daily and indomethacin 50 mg three times daily			1	1	2	No improvement in gout flare. Acute kidney injury developed in both patients.
SCHLONDORFF 1993 [86]	Case report	Creatinine clearance of 70 (serum creatinine of 1.8 mg/dL)	Gout flare treatment	Indomethacin 50 mg three times daily		1			1	Gout flare improved rapidly. Acute kidney injury ensued.
ZAGLER 2009 [57]	Case report	eGFR of 34	Gout flare treatment	Diclofenac 100 mg/d			1		1	No improvement in gout flare. However, renal function deteriorated (serum creatinine increased from 2.4 to 5.2 mg/dL).
GLUCOCORTICOIDS										
TAUSCHE 2011 [95]	Case report	eGFR of 30	Gout flare prophylaxis	Prednisone 10 mg/d			1		1	Improvement in frequency of gout flare (in combination with regular low-dose colchicine use and low-dose NSAID as required; the duration for the gout flare prophylaxis use was not known). Renal function remained stable.
ZAGLER 2009 [57]	Case report	eGFR of 34	Gout flare treatment	Prednisone 40 mg/d			1		1	Gout flare treatment was effective. However, renal function deteriorated (serum creatinine increased from 2.4 to 5.2 mg/dL).

*AKI* acute kidney injury, *CKD* chronic kidney disease, *CRP* C-reactive protein, *CrCl* creatinine clearance, *eGFR* estimated glomerular filtration rate, *ESRD* end-stage renal disease, *hsCRP* highly sensitive C-reactive protein, *IV* intravenous, *PO* per os (by mouth), *VAS* visual analogue score

### Colchicine

A total of 2325 studies of colchicine use were identified, as summarised in a PRISMA flowchart of the literature search (Supplementary Figure 1), and a final total of 49 studies were eligible for data extraction [9–57]. Twenty of these 49 studies, which were mostly case series or case reports, described the efficacy and/or safety outcomes of colchicine use stratified by renal function, as summarised in Tables 2 and 3, respectively [17, 18, 20, 22, 24, 28, 29, 31–33, 35–37, 39, 44, 46, 49, 53, 55, 57]. The remaining 29 studies reported efficacy and/or safety outcomes of colchicine use without renal function stratification, as summarised in Supplementary Table 2 and Supplementary Table 3, respectively [9–16, 19, 21, 23, 25–27, 30, 34, 38, 40–43, 45, 47, 48, 50–52, 54, 56].

**Table 3:** Safety outcome reporting of gout flare prophylaxis and therapy use with renal function stratification

First Author (Year) (Trial Name)	Adverse/Serious Adverse Events Reported by Renal Function	Notable Findings
COLCHICINE		
AKDAG 2006 [17]	Yes	Colchicine toxicity in the context of antibiotic use (clarithromycin and cefepime) for pneumonia. Besides worsening renal function, there was associated mild pancytopaenia and liver impairment.
ALAYLI 2005 [18]	Yes	Colchicine neuromyopathy in the context of concomitant statin use.
ALTMAN 2007 [20]	Yes	Colchicine-induced rhabdomyolysis.
BONNEL 2002 [22]	Yes	Fatal colchicine toxicity with rapidly deteriorating renal function and death.
BOUQUIÉ 2011 [24]	Yes	Colchicine-induced rhabdomyolysis with acute decompensation of pre-existing double heart/lung transplant and multi-organ failure.
ELEFTHERIOU 2008 [28]	Yes	Colchicine toxicity with multi-organ failure in the context of concomitant long-term cyclosporin therapy for heart transplant.
GARROUSTE 2012 [29]	Yes	Colchicine toxicity with neuromyopathy and multi-organ failure in the context of concomitant long-term cyclosporin use for renal transplant.
HUH 2013 [31]	Yes	Colchicine toxicity with gastrointestinal symptoms and neuromyopathy in the context of concomitant long-term cyclosporin use for renal transplant and statin use.
JUSTINIANO 2007 [32]	Yes	Colchicine-induced rhabdomyolysis in the context of concomitant statin use.
KUBLER 2000 [33]	Yes	Fatal colchicine toxicity with multi-organ failure and death.
LAI 2006 [35]	Yes	Colchicine neuromyopathy.
LEE 1997 [36]	Yes	Acute myopathy in the context of concomitant cyclosporin use for renal transplant.
LY 2007 [37]	Yes	One patient with CKD (serum creatinine of 0.21 mmol/L) developed colchicine myopathy.
MEDANI 2016 [39]	Yes	Colchicine neuromyopathy.
NEUSS 1986 [44]	Yes	Fatal colchicine toxicity with myopathy, multi-organ failure, severe neutropaenia with associated disseminated candidiasis and death.
PATEL 2016 [46]	Yes	Colchicine-induced rhabdomyolysis in the context of statin use and initiation of sofosbuvir/ledipasvir therapy for Hepatitis C.
RIEGER 1990 [49]	Yes	Colchicine neuromyopathy in the context of acute stage of post-renal transplant and cyclosporin use.
WILBUR 2004 [53]	Yes	Colchicine neuromyopathy.
YOON 2001 [55]	Yes	Colchicine toxicity with associated pancytopaenia, neuromyopathy and total alopecia.
ZAGLER 2009 [57]	Yes	Colonic perforation and acute on chronic CKD.
IL-1 INHIBITORS		
ADLER 2007 [67]	No	Anakinra treatment: adverse event was not reported.
AOUBA 2015 [68]	Yes	Anakinra treatment: 1 patient with mild injection site reaction, transient diffuse pruritus and episodic diarrhoea.
BARTOV 2013 [69]	Yes	Anakinra treatment: adverse event was not observed.
DIREZ 2012 [71]	Yes	Anakinra treatment: non-complicated neutropaenia.
LOUSTAU 2018 [76]	Yes	Anakinra treatment: 1 patient with an infection (nosocomial pyelonephritis). No other adverse event was observed.
MAROTTO 2018 [77]	Yes	Canakinumab treatment: adverse event was not observed.
PEREZ-RUIZ 2013 [81]	Yes	Anakinra treatment: 1 patient with recurrent heart failure. No other adverse event was observed.
TRAN 2011 [83]	Yes	Anakinra treatment: adverse event was not reported.
NON-STEROIDAL ANTI-INFLAMMATORY DRUGS		
KAHL 1989 [84]	Yes	Acute kidney injury with sulindac and indomethacin use.
SCHLONDORFF 1993 [86]	Yes	Acute kidney injury with indomethacin use.
ZAGLER 2009 [57]	Yes	Colonic perforation and acute on chronic CKD.
GLUCOCORTICOIDS		
TAUSCHE 2011 [95]	No	Adverse event was not reported.
ZAGLER 2009 [57]	Yes	Colonic perforation and acute on chronic CKD.

*CKD* Chronic kidney disease

#### Studies of colchicine use with analyses stratified by renal function

These 20 studies included 1 single-centre audit, 3 case series, and 16 case reports, with a total of 43 study participants. Varying patterns in colchicine dosing amount and frequency as well as varying routes of drug administration were seen across these studies. Only 5 studies reported efficacy outcome data stratified by renal function, although no definitive conclusion could be drawn due to their heterogenous study characteristics and the quality of the evidence was low [18, 33, 44, 53, 55].

For the safety outcome, all studies reported varying level of transient change in renal function during colchicine use as well as after drug cessation. Fifteen studies reported colchicine-induced neuromyopathy and rhabdomyolysis [18, 20, 24, 29, 31, 32, 35–37, 39, 44, 46, 49, 53, 55]. Nine studies reported colchicine toxicity secondary to drug-drug interaction with statin, cyclosporin, clarithromycin, and hepatitis C treatments (i.e. sofosbuvir/ledipasvir) [17, 18, 28, 29, 31, 32, 36, 46, 49]. It was unclear from these studies if the reported adverse events were directly attributed to the colchicine use.

#### Studies of colchicine use without analyses stratified by renal function

These 29 studies included 2 single-centre randomised controlled trials (RCTs), 1 post hoc analysis of 3 different ULT-related RCTs, 1 post hoc analysis of a cross-sectional study, one case-control study, 2 retrospective observational studies, 4 audits, 8 case series, and 10 case reports. All studies included study participants with varying degree of baseline renal impairment, and only pooled efficacy and safety outcome data were presented, without any renal function stratification in the outcome reporting. Furthermore, in studies using colchicine as gout flare prophylaxis, study participants with eGFR of < 30 mL/min/1.73 m^2^ were excluded, as evident in the RCT by Borstad and colleagues and the post hoc study of 3 different ULT-related RCTs [10, 11].

### IL-1 inhibitors

A total of 1067 studies of IL-1 inhibitor use were identified, as summarised in a PRISMA flowchart of the literature search (Supplementary Figure 2), and a final total of 26 studies were eligible for data extraction [58–83]. Eight of these 26 studies, which were mostly case series or case reports, described the efficacy and/or safety outcomes of IL-1 inhibitor use stratified by renal function, as summarised in Tables 2 and 3, respectively [67–69, 71, 76, 77, 81, 83]. The remaining 18 studies reported efficacy and/or safety outcomes of IL-1 inhibitor use without renal function stratification, as summarised in Supplementary Table 2 and Supplementary Table 3 respectively [58–66, 70, 72–75, 78–80, 82]. Overall, there were 17 studies of anakinra use, 7 studies of canakinumab use, and 2 studies of rilonacept use.

#### Studies of IL-1 inhibitor use with analyses stratified by renal function

These 8 studies of IL-1 inhibitor use as gout flare therapy included 4 case series and 3 case reports of anakinra use, and 1 case report of canakinumab use. A standard 100-mg dosing was routinely observed in studies of anakinra use, albeit varying dose frequency and duration in the context of varying degree of renal impairment and the duration of gout flare. Six out of 7 studies of anakinra use demonstrated stable renal function during treatment irrespective of pre-existing CKD [67–69, 76, 81, 83]. One case report of anakinra use described a decline in renal function [71]. In terms of anakinra’s safety profile, 4 studies reported non-fatal infection-related adverse events [68, 71, 76, 81]. The case report on canakinumab use described good efficacy in treating gout flare without any compromise in renal function or in safety signal [77]. No definitive conclusion on IL-1 inhibitor use in CKD could be drawn due to the low quality of evidence for these studies.

#### Studies of IL-1 inhibitor use without analyses stratified by renal function

There were 10 studies of anakinra use (1 single-centre open-label clinical trial, 4 case series, and 5 case reports) [60, 70, 72–75, 78–80, 82]. In the open-label clinical trial of anakinra use by So and colleagues, study participants with advanced CKD (i.e. eGFR of < 30 mL/min/1.73 m^2^) were excluded from the study and the efficacy and/or safety outcomes for CKD subgroups were not presented, as only pooled results were reported [60].

All RCTs of canakinumab use, described in 6 different published articles, excluded individuals with advanced CKD (i.e. eGFR of < 30 mL/min/1.73 m^2^) [58, 59, 61–64]. These studies included a multi-centre phase 2 trial evaluating the efficacy of canakinumab of varying doses (with the initial study results reported by So and colleagues, followed by the remaining study results reported by Schlesinger and colleagues) [58, 61], and the β-RELIEVED and β-RELIEVED II randomised trials (with study results reported in 3 separate published articles) [59, 62, 63]. In the β-RELIEVED and β-RELIEVED II randomised trials, although the analyses were performed on a subgroup of participants with CKD ≥ stage 3, pooled outcome results were presented [59, 62, 63]. Similarly, for the β-RELIEVED and β-RELIEVED II randomised trials looking at different canakinumab formulations, pooled outcome results for CKD subgroup were presented [64].

Two clinical trials of rilonacept use (1 crossover trial and 1 post hoc analysis of PRE-SURGE 1, PRE-SURGE 2, and RE-SURGE clinical trials) also similarly excluded study participants with CKD ≥ stage 3 and only pooled outcome results were presented [65, 66].

### NSAIDs

Using the search terms as outlined in Supplementary Table 1, 1835 studies of NSAID use were initially identified, as summarised in a PRISMA flowchart summary (Supplementary Figure 3). After a sequential review of the title, abstract, and full-text, a final total of 4 studies of NSAID use were included for data extraction, with 3 studies reported efficacy and/or safety outcomes stratified by renal function and the remaining 1 study had study outcomes reported without renal function stratification [57, 84–86]. These 4 studies largely aimed at showcasing the potential risk for nephrotoxicity with NSAID use as gout flare prophylaxis and therapy in patients with CKD.

#### Studies of NSAID use with analyses stratified by renal function

There were 1 case series and 2 case reports of NSAID use reporting study outcomes based on renal function (Tables 2 and 3) [57, 84, 86]. Of note, these studies documented the onset of acute kidney injury (AKI) with NSAID use in patients with gout flare and concomitant CKD. However, despite the eligibility for data extraction, these studies had insufficient number of patients and information to accurately ascertain the efficacy or toxicity of NSAID use in managing gout flares in patients with CKD.

#### Studies of NSAID use without analyses stratified by renal function

One case series, although without outcome results documented by renal function stratification, described an association between NSAID use and risk of developing AKI (Supplementary Table 2 and Supplementary Table 3) [85].

### Glucocorticoids

Using the search terms as outlined in Supplementary Table 1, 1678 studies of glucocorticoid use were initially identified, as summarised in a PRISMA flowchart summary (Supplementary Figure 4). After a sequential review of the title, abstract and full-text, a final total of 12 studies were included for data extraction [57, 64, 87–96]. Eleven out of these 12 studies were of case reports (*n* = 10) and of case series (*n* = 1) [57, 87–96]. Therefore, the evidence provided from these limited data did not allow any accurate conclusion drawn on the efficacy and/or safety of glucocorticoid use in gout flare and concomitant CKD. Only 2 studies reported efficacy and/or safety outcomes stratified by renal function and the remaining 10 study had study outcomes reported without renal function stratification.

#### Studies of glucocorticoid use with analyses stratified by renal function

Two case reports had outcome results stratified by renal function, but further conclusion could not be drawn due to the low level of evidence for these studies (Tables 2 and 3) [57, 95].

#### Studies of glucocorticoid use without analyses stratified by renal function

Ten studies of glucocorticoid use described outcome results without renal function stratification (Supplementary Table 2 and Supplementary Table 3) [64, 87–94, 96]. One single-centre case series by Bajaj and colleagues described a cohort of 10 lupus patients with gout flare, of which 8 of them were on varying doses of prednisone as gout flare therapy [87].

## Discussion

This review explores the current literature on the efficacy and safety outcome data on the use of gout flare prophylaxis and therapy in people with CKD ≥ stage 3. Without limiting the publication date and study design, we were able to capture all of the efficacy and/or safety data for different anti-inflammatory therapy used for gout flare in people with underlying renal impairment. Using the best evidence synthesis approach, we then extracted and summarised the outcome data for each study based on the presence or absence of renal function stratification. Overarchingly, this review has highlighted the absence of conclusive data on efficacy or safety in gout flare prophylaxis and therapy use in patients with underlying advanced CKD.

Although colchicine has been used for many years and remains a first-line anti-inflammatory drug for gout flare prophylaxis and therapy, we currently have insufficient data to adequately inform us on the efficacy and safety of using colchicine in people with gout and concomitant CKD. For instance, there are only 2 single-centre RCTs and 1 post hoc analysis study of three RCTs reporting on colchicine prophylactic use in people with underlying CKD, although these clinical trials presented aggregated outcome results (i.e. without renal function stratification) for the efficacy and safety data on colchicine use in this subgroup, and these results are not necessarily informative for people with varying CKD stages. In addition, we have seen different results on the impact of gout flare treatment on renal function in case reports and case series. For instance, 12 studies reported deteriorated renal function with colchicine use [17, 20, 22, 24, 32, 33, 35, 39, 44, 53, 55, 57], whereas 7 other studies reported stable renal function with colchicine use [18, 28, 29, 31, 36, 46, 53]. As a result, given the underlying risk of bias on study quality for these studies, we cannot sufficiently conclude on the efficacy and/or safety outcomes on colchicine use for people with gout and concomitant CKD. In the AGREE clinical trial, low-dose colchicine use was as comparably effective as the high-dose colchicine in gout flare with minimal side effects, and therefore, low-dose colchicine has been recommended for use in gout flare prophylaxis and therapy [97]. The question remains, whether low-dose colchicine use remains effective and safe, for treatment of flares or flare prophylaxis, in those with advanced CKD. Similarly, we do not have adequate efficacy and safety outcome data for IL-1 inhibitor use in gout flare and CKD to inform clinicians if renally adjusted dosing is required when using these IL-1 inhibitors for different renal disease severity. The issue of IL-1 inhibition use for flare prophylaxis in patients with gout and advanced CKD remains essentially unexplored. Additionally, from the pharmacovigilance perspective, drug tolerance is an important consideration when using these anti-inflammatory medications in gout flare. For colchicine, increased drug toxicity is seen in individuals with CKD, due to increased drug half-life. In addition, the overall colchicine-related side effects secondary to drug retention are more noticeable when treating gout flare transiently in the clinical settings of concomitant CKD and acute illness such as dehydration and sepsis. It is also important to note that colchicine use in gout and advanced CKD can be hazardous when used in conjunction with some medications, such as statin therapies (CYP3A4 inhibitors), cyclosporin (both CYP3A4 and P-glycoprotein inhibitors), and macrolide antibiotic, such as clarithromycin (both CYP3A4 and P-glycoprotein inhibitors). Similarly, for anakinra, the dose should be renally adjusted in individuals with gout and advanced CKD due to the risk of increased drug half-life, and yet, this recommendation is seldom applied in the clinical practice.

In the case of NSAID use as gout flare prophylaxis and therapy, we did not expect to find any recent data to justify NSAID use in CKD, as all NSAIDs are widely known to be contraindicated in advanced CKD. Indeed, the included case series/reports of NSAID use in this review favourably justify the avoidance of any NSAID use in individuals with gout and renal failure. Almost all studies found were only aiming at highlighting the nephrotoxic risk of NSAID use in this high-risk comorbid population with gout flare. The question remains, however, as to whether NSAID use is equally effective and safe in patients with non-residual renal function compared with those with normal renal function but we did not find any published evidence to support or refute that hypothesis. In the case of glucocorticoid use, all studies found described either refractory or very severe gout flare cases, which are not necessarily reflecting the common clinical practice of gout flare management. We did not find studies exploring the question of whether low doses of glucocorticoids could be part of the prophylaxis of gout flares. Another question that remains is whether glucocorticoid use is equally effective and safe or if there is a potential risk of exacerbating tophaceous gout disease.

Furthermore, we found that all clinical trials reported pooled data on efficacy and/or safety outcomes, even with renal function stratified at baseline for all study participants. Pertinent to our review aims, it is evident that most clinical trials of gout flare prophylaxis and therapy excluded study participants with advanced CKD (i.e. CKD ≥ stage 3). This is likely explained by the strict regulations implemented in most clinical trial approval by the US Food and Drug Administration (FDA) and the European Medicines Agency (EMA), of which these regulatory bodies restrict the inclusion of study participants with CKD ≥ stage 3. In terms of profiling drug safety in the management of gout flare, we identified certain side effects being reported in the studies, but unlikely to have any attribution to the underlying renal impairment. For example, infections were commonly reported for IL-1 inhibitors and glucocorticoid use, which would be likely due to the immunomodulatory effects from the drug use, rather than the effects of the underlying reduced renal function. This finding highlights the importance of profiling drug safety with the comparison between individuals with and without CKD in gout studies, where possible.

This review has highlighted the heterogenous patterns in efficacy and/or safety outcome reporting in all studies on gout flare management and prophylaxis, irrespective of the study designs. This observation is also echoed by a recent systematic review by Stewart and colleagues on gout flare reporting in clinical trials [98]. Besides the patient’s self-reported gout flare resolution and other symptom reporting, objective assessments such as using the pain visual analogue score (VAS) and C-reactive protein (CRP) level are commonly implemented in the study protocols in evaluating treatment efficacy in most clinical trials and observational studies of gout flare management. Yet, these objective assessments are not necessarily standardised among clinical trials and the gout flare definition may differ between studies. Such issues can further complicate the interpretation of study findings when comparisons between studies are made collectively. A recent validation study in defining gout flare by Gaffo and colleagues has stressed the importance of having an accurate and validated definition and assessment of gout flare in all clinical studies of gout [99]. By incorporating standardised gout flare definition in future gout flare studies, comparisons in treatment outcomes across studies of different treatments used as gout flare prophylaxis and therapy can be performed fairly and efficiently. Ideally, the efficacy and safety of gout flare and urate-lowering treatments based on stratified renal function should be emphasised in all gout studies, as gout and CKD often co-exist. For example, an ongoing Veterans Affairs (VA) StopGOUT study in the USA is currently evaluating the ‘treat-to-target’ dose escalation of urate-lowering therapies (allopurinol versus febuxostat) in managing gout and with further observation in assessing the efficacy and safety of renally adjusted dosing in study participants with co-existing CKD [100].

This review has some study limitations. We did not include non-English published studies or unpublished data, which could potentially lead to bias in the study inclusion and exclusion process. Specifically, relevant information on the use of IL-1 inhibitors may be missed, considering that anakinra is an off-label use for gout flare therapy in some countries and canakinumab is not widely indicated for gout flare therapy in some English-speaking countries. We did not have sufficient data for people with gout flare and underlying renal transplant, and therefore, the findings from this review may not reflect on this specific renal disease subgroup. Due to the heterogeneity nature of the study designs across all included studies, quantitative analysis such as meta-analysis could not be performed. In general, we propose that the overall findings and interpretations of this review using the best evidence synthesis approach is unlikely to differ despite our study limitations.

## Conclusion

In summary, this review has described the current literature on the efficacy and safety of gout flare prophylaxis and therapy use in people with gout and concomitant CKD. The dearth of high-quality data reporting in this high-risk comorbid population is concerning, especially in clinical trials. Currently, treating clinicians have no evidence-based approaches to manage flares or prophylaxis in patients with gout and advanced CKD. Current and future gout flare studies should include patients with CKD and inform study results stratified by renal function as well as using standardised gout flare definitions in the study design. With these key steps, results of future gout flare prophylaxis and treatment studies will guide better and systematic evidence-informed approach in managing gout flares and prophylaxis in patients with advanced CKD.

## Supplementary Information

**Additional file 1.**

## Data Availability

All data are in the manuscript and supplementary files.

## Abbreviations

ACR: American College of Rheumatology
AKI: Acute kidney injury
CKD: Chronic kidney disease
CrCL: Creatinine clearance
CRP: C-reactive protein
eGFR: Estimated glomerular filtration rate
EMA: European Medicines Agency
ESRD: End-stage renal disease
EULAR: European League Against Rheumatism
FDA: Federal Drug Administration
G-CAN: Gout, Hyperuricemia and Crystal-Associated Disease Network
IL: Interleukin
NSAIDs: Non-steroidal anti-inflammatory drugs
PRISMA: Preferred Reporting Items for systematic Reviews and Meta-Analyses
RCTs: Randomised controlled trials
ULT: Urate-lowering therapy
VAS: Visual analogue scale
