# Supplementary material for: Efficacy and safety of gout flare prophylaxis and therapy use in people with chronic kidney disease: a Gout, Hyperuricemia and Crystal-Associated Disease Network (G-CAN)-initiated literature review
Source: Arthritis Res Ther. 2021 Apr 28;23:130. doi: 10.1186/s13075-021-02416-y (PMC8080370; doi:10.1186/s13075-021-02416-y)
Supplement: Supplementary file 1 — Additional file 1. [file 13075_2021_2416_MOESM1_ESM.docx]

**Supplementary Materials**

**Supplementary Figures**

**Supplementary Figure 1: PRISMA flowchart for the study eligibility for colchicine**

Additional records identified through other source
(n = 0)

Records identified through database searching
(n = 2325)

## Identification

Records excluded (n = 1680)

- Review/opinion/editorial n = 604
- Laboratory/in vitro n = 63
- Not related to study drug n = 466
- Not related to gout n = 444
- Qualitative studies n = 10
- Full-text article not found n = 74
- Miscellaneous n = 19

Records after duplicates removed
(n = 2096) (229 duplicates)

## Screening

Records screened
(n = 2096)

Full-text articles excluded (n = 365)

- Review/editorial/opinion n = 36
- Normal renal function or CKD Stage 1-2 n = 49
- Acute renal failure n = 12
- No CKD information n = 183
- Not related to study drug n = 61
- Not related to gout n = 13
- Full-text article in foreign language n = 1
- Miscellaneous n = 12

Full-text articles assessed for eligibility
(n = 416)

## Eligibility

Data extraction

- Studies with sub-analysis based on GFR n = 20
- Studies without sub-analysis based on GFR n = 29

## Included

Studies included

(n = 49)

CKD: chronic kidney disease; GFR: glomerular filtration rate; PRISMA: Preferred Reporting Items for Systematic Reviews and Meta-Analyses

**Supplementary Figure 2: PRISMA flowchart for the study eligibility for Interleukin-1 (IL-1) inhibitors**

## Identification

Records identified through database searching
(n = 1067)

Additional records identified through other source
(n = 0)

Data extraction

- Studies with sub-analysis based on GFR n = 8
- Studies without sub-analysis based on GFR n = 18

Studies included

(n = 26)

## Included

## Eligibility

Full-text articles assessed for eligibility
(n = 88)

Full-text articles excluded (n = 62)

- Editorial n = 1
- Laboratory/in vitro n = 1
- Normal renal function or CKD Stage 1-2 n = 6
- No CKD information n = 51
- Not related to study drug n = 1
- Not related to gout n = 1
- Miscellaneous n = 1

Records excluded (n = 835)

- Review/opinion/editorial n = 133
- Laboratory/in vitro n = 34
- Not related to study drug n = 48
- Not related to gout n = 610
- Miscellaneous n = 10

Records after duplicates removed
(n = 923) (144 duplicates)

Records screened
(n = 923)

## Screening

CKD: chronic kidney disease; GFR: glomerular filtration rate; PRISMA: Preferred Reporting Items for Systematic Reviews and Meta-Analyses

**Supplementary Figure 3: PRISMA flowchart for the study eligibility for non-steroidal anti-inflammatory drugs (NSAIDs)**

**Included**

Data extraction

- Studies with sub-analysis based on GFR n = 3
- Studies without sub-analysis based on GFR n = 1

Studies included
(n = 4)

Full-text articles excluded (n = 397)

- Animal studies n = 1
- Basic science of purely lab n = 3
- Review articles n = 53
- Editorial n = 2
- Opinion based papers n = 4
- Papers not pertinent to study drug n = 114
- Duplicates n = 7
- Not about gout n = 73
- No data on CKD n = 131
- Full manuscript not available n = 10

**Eligibility**

Full-text articles assessed for eligibility
(n = 402)

**Screening**

Records screened
(n = 1824)

Records excluded (n = 1422)

- Animal studies n = 8
- Basic science or purely lab n = 75
- Review articles n = 377
- Editorial n = 85
  Opinion based papers n = 877

Records after duplicates removed
(n = 1824) (11 duplicates)

Additional records identified through other sources
(n = 0)

Records identified through database searches
(n = 1835)

**Identification**

CKD: chronic kidney disease; GFR: glomerular filtration rate; PRISMA: Preferred Reporting Items for Systematic Reviews and Meta-Analyses

**Supplementary Figure 4: PRISMA flowchart for the study eligibility for glucocorticoids**

Additional records identified through other sources
(n = 0)

Records identified through database searches
(n = 678)

**Identification**

Records excluded (n = 463)

- Basic science n = 23
- Review article n = 234
- Editorial n = 36
- Opinion based n = 170

Records after duplicates removed
(n = 2) (2 duplicates)

**Screening**

Records screened
(n = 676)

Full-text articles excluded (n = 200)

- Review article n = 6
- Does not pertain to drug n = 83
- Duplicate n = 2
- Not about gout n = 3
- No information on GFR n = 106
- No access to full text n = 1

Full-text articles assessed for eligibility
(n = 213)

**Eligibility**

Data extraction

- Studies with sub-analysis based on GFR n = 2
- Studies without sub-analysis based on GFR n = 10

Studies included
(n = 12)

**Included**

CKD: chronic kidney disease; GFR: glomerular filtration rate; PRISMA: Preferred Reporting Items for Systematic Reviews and Meta-Analyses

**Supplementary Tables**

**Supplementary Table 1: Search terms**

| Search strategy 1 | "Gout"[Mesh] OR gout [tiab] OR gouty-arthritis [tiab] | AND "Anti-Inflammatory Agents, Non-Steroidal"[Mesh] OR “Non-Steroidal Anti-Inflammatory Agents” [tiab] OR “NonSteroidal Anti-Inflammatory Agents” [tiab] OR NSAID [tiab] OR NSAIDs [tiab] OR "Colchicine"[Mesh] OR Colchicine [tiab] OR "Adrenal Cortex Hormones"[Mesh] OR Corticosteroids [tiab] OR glucocorticoids [tiab] OR "Steroids"[Mesh] OR steroids [tiab] OR prednisone [tiab] OR prednisolone [tiab] OR "canakinumab" [Supplementary Concept] OR canakinumab [tiab] OR "Interleukin 1 Receptor Antagonist Protein"[Mesh] OR Anakinra [tiab] | NOT ("animals"[MeSH Terms]NOT "humans"[MeSH Terms]) | AND English [la] |
| --- | --- | --- | --- | --- |
| Search Strategy 2 | "Renal Dialysis"[Mesh] OR renal-dialys* [tiab] OR kidney-dialys* [tiab] OR extracorporeal-dialys* [tiab] OR peritoneal-dialys* [tiab] OR hemodialys* [tiab] OR hemodiafiltration [tiab] OR "Renal Insufficiency, Chronic"[Mesh] OR ckd [tf] OR chronic-renal-insufficienc* [tiab] OR chronic-kidney-insufficienc* [tiab] OR “chronic kidney disease” [tiab] OR “chronic kidney diseases” [tiab] OR “chronic renal disease” [tiab] OR “chronic renal diseases” [tiab] OR “chronic renal failure” [tiab] OR “chronic kidney failure” [tiab] OR esrd [tf] OR “end stage renal failure” [tiab] OR “end stage kidney failure” [tiab] OR “end stage renal disease” [tiab] OR “end stage kidney disease” [tiab] | AND "Anti-Inflammatory Agents, Non-Steroidal"[Mesh] OR “Non-Steroidal Anti-Inflammatory Agents” [tiab] OR “NonSteroidal Anti-Inflammatory Agents” [tiab] OR NSAID [tiab] OR NSAIDs [tiab] OR "Colchicine"[Mesh] OR Colchicine [tiab] OR "canakinumab" [Supplementary Concept] OR canakinumab [tiab] OR "Interleukin 1 Receptor Antagonist Protein"[Mesh] OR Anakinra [tiab] | NOT ("animals"[MeSH Terms] NOT "humans"[MeSH Terms]) | AND English [la] |

**Supplementary Table 2: Efficacy outcome reporting of gout flare prophylaxis and therapy use without renal function stratification**

| First Author (Year)  (Trial Name) | Study Design | Renal Function Exclusion Criteria or Baseline Renal Function – eGFR/CrCl (mL/min/1.73m2) or Serum Creatinine Level | Clinical Indication for Gout Flare | Actual/Mean Gout Flare Therapy Dose | Number of Participants by eGFR/CrCl at Baseline (mL/min/1.73m^2^) | | | | Total, n | Efficacy Data without Renal Function Stratification |
| --- | --- | --- | --- | --- | --- | --- | --- | --- | --- | --- |
|  |  |  |  |  | **≥90** | **60-90** | **30-60** | **<30** |  |  |
| COLCHICINE | | | | | | | | | | |
| AHERN 1987(1) | RCT (single-centre) | Mean serum creatinine of 140±8µmol/l | Gout flare treatment | Initial dose of 1mg PO, followed by 0.5mg PO every 2 hours until clinical response or toxicity  Mean dose of 6.7mg | 22 | | | | 22 | Pooled efficacy outcome: 64% in the colchicine arm achieved gout flare improvement clinically 48 hours post-treatment (vs 23% in the placebo arm) and 73% in colchicine arm reported improved pain VAS 48 hours post-treatment (vs 36% in the placebo arm). |
| BORSTAD 2004(2) | RCT (single-centre) | Study participants with CrCl of <20mL/min were excluded. | Gout flare prophylaxis | 0.6mg PO once daily | 14 | | |  | 14 | Pooled efficacy outcome: in the colchicine arm, there were fewer total flares (fewer flares in the first 3 months and beyond, up to 6 months), less severe flares based on pain VAS (3.64 vs 5.08 in the placebo arm) and less recurrent gout flares. |
| WORTMANN 2010  (FACT, APEX, CONFIRMS)(3) | Three RCTs (post-hoc analyses) | Study participants with CrCl of <30 were excluded in all of these trials. | Gout flare prophylaxis | 0.6mg PO once daily | 371(F); 541(A); 786(C) | 295(F); 154(A); 402(C) | 94(F); 154(A); 402(C) |  | 760(F); 1072(A); 2269(C) | Pooled efficacy outcome: less flare rates for those using colchicine for 6 months, compared to those on the 8-week duration. |
| PASCART 2016 (GOSPEL 2)(4) | Cross-sectional study (post-hoc analysis) | Patients with different CKD stages were all included. | Gout flare treatment | Overall mean dose 2.8±0.7mg; overall cumulative dose over the first 3 days 6.9±1.8mg (range 3-18mg)  Normal renal function: mean daily dose 2.9±0.6mg  CKD Stage 2: 2.8±0.8mg  CKD Stage 3: 2.7±0.8mg  CKD Stage 4: 2.5±0.7mg | 158 | 59 | 45 | 2 | 264 | 79% of patients achieved pain relief by day 7 (0.8±1.0cm on a 0-6 numeric pain scale, compared to baseline average pain of 4.9±0.9cm). |
| SOLAK 2014(5) | Case-control | ESRD with eGFR of <30 (on haemodialysis) | Gout flare treatment | 0.5-1.5mg PO daily |  |  |  | 1 | 1 | Efficacy data not available. |
| HUNG 2005(6) | Retrospective observational (single-centre) | Study participants with CKD (serum creatinine of >140 µmol/l) were included. | Gout flare treatment | Total median dose: 3.0mg (range 1.63-4.5) in the concomitant use arm (clarithromycin) vs 3.5mg (range 2.63-5) in the sequential use arm (clarithromycin) | 29 (concomitant arm) vs 8 (sequential arm) | | | | 37 | Efficacy data not available. |
| KWON 2017(7) | Retrospective observational (single-centre) | Study patients with CKD of any stages were included. | Gout flare treatment or prophylaxis | 0.88±0.29mg/d (colchicine with statin arm) vs 0.96±0.27mg/d (colchicine without statin arm) | 36/188 (colchicine with statin arm) vs 41/486 (colchicine without statin arm) | | | | 77 | Efficacy outcome was not assessed. |
| AKAR 2001(8) | Case report | eGFR of 17 (serum creatinine of 3.88mg/dL) | Gout flare prophylaxis | 0.5mg PO three times daily (for at least 10 years) |  |  |  | 1 | 1 | Efficacy data not available. |
| ALTIPARMAK 2002(9) | Case series | ESRD with eGFR of 6 (serum creatinine of 9.4mg/dL) | Gout flare prophylaxis | 0.5mg PO three times daily (for at least 15 years) |  |  |  | 1 | 1 | Efficacy data not available. |
| BAKER 2004(10) | Case report | eGFR of 30 (serum creatinine of 200µmol/l) | Gout flare treatment | 0.6mg PO daily for 4 days, then increased to 0.6mg PO twice daily (persistent flare) |  |  | 1 |  | 1 | Efficacy data not available. |
| BOOMERSHINE 2002(11) | Case report | ESRD on peritoneal dialysis with eGFR of 4 (serum creatinine of 15.5mg/dL) | Gout flare treatment | 0.6mg PO twice daily (for at least 2 weeks) |  |  |  | 1 | 1 | Efficacy data not available. |
| CHENG 2005(12) | Case series | ESRD on peritoneal dialysis with eGFR of 9 (serum creatinine of 484µmol/l) | Gout flare treatment | 0.5mg PO three times daily |  |  |  | 1 | 1 | Efficacy data not available. |
| CHOI 1999(13) | Case report | eGFR of 29 (serum creatinine of 178µmol/l) | Gout flare treatment | 0.5mg PO twice daily (for at least 4 weeks) |  |  |  | 1 | 1 | Efficacy data not available. |
| DIXON 2001(14) | Case report | eGFR of 35 (serum creatinine of 2.0mg/dL) | Gout flare prophylaxis and treatment | 0.6mg PO  twice daily (for at least 3 years), followed by 0.6mg PO every 4 hours for gout flare |  |  | 1 |  | 1 | Efficacy data not available. |
| HSU 2002(15) | Case report | eGFR of 22 (serum creatinine of 3.0mg/dL) | Gout flare treatment | 0.5mg PO twice daily (for at least 3 weeks) |  |  |  | 1 | 1 | Efficacy data not available. |
| KUNCL 1987(16) | Case series | CKD (serum creatinine of ≥1.6mg/dL) | Gout flare prophylaxis |  | 12 | | | | 12 | Efficacy data not available. |
| MARCINIAK 2016(17) | Case report | eGFR of 43 (serum creatinine of 1.6mg/dL) | Gout flare treatment | 0.6mg PO once daily (for at least 2 years) |  |  | 1 |  | 1 | Efficacy data not available. |
| MONTSENY 1996(18) | Case series | eGFR of <30 (range 7-26; serum creatinine of 249-542µmol/l) | Gout flare treatment | 1mg PO once daily (duration range 7-14 days) |  |  |  | 4 | 4 | Efficacy data not available. |
| MORRIS 2003(19) | Case series | eGFR of 18 (serum creatinine of 236µmol/l) | Gout flare treatment | Initial dose of 1mg PO, followed by 0.5mg every 8 hours (patient developed GI symptoms). After 24 hours, patient was resumed on 0.5mg PO twice daily dosing. |  |  |  | 1 | 1 | Efficacy data not available. |
| MULLINS 2011(20) | Audit (single-centre) | Variable CrCl (range 11-132) | Gout flare treatment | Varying in dose and administrative route (1.2mg/d for CrCl ≥50, 0.6mg/d for CrCl 10-50) | 4 | 7 | 16 | 10 | 37 | Efficacy data not available. |
| NASHEL 1982(21) | Case series | eGFR of <30 (serum creatinine of 2.9-4.8mg/dL) | Gout flare treatment | Patient 1: initial dose of 2mg IV, followed by 1mg IV every 8 hours (for at least 24 hours)  Patient 2: 0.6mg IV twice daily |  |  |  | 2 | 2 | Gout flare resolution was achieved for both patients (patient 1 – within 24 hours). |
| ORTEL 1974(22) | Case report | eGFR of 31 (serum creatinine of 2.7mg/dL) | Gout flare treatment | 0.5mg PO hourly dosing - total of 4.5mg for the first gout flare, and total of 3.5mg for the second gout flare |  |  | 1 |  | 1 | Gout flare resolution was achieved in both episodes. |
| PETERSEL 2007(23) | Audit (single-centre) | Study patients with CKD of any stages were included. | Gout flare treatment | Thirty patients with CKD received 0.6mg PO three times daily and the remaining patients with CKD had 0.6mg PO every hour. | 38 | | | | 38 | Efficacy data not available. |
| RANA 1997(24) | Case series | eGFR range 24-39 (serum creatinine range 1.9-2.9mg/dL) | Gout flare prophylaxis | 0.6mg PO daily |  |  | 3 | | 3 | Efficacy data not available. |
| RUTKOVE 1996(25) | Case series | eGFR of <30 | Gout flare prophylaxis | 0.6mg PO daily (duration range 9-18 months) |  |  |  | 4 | 4 | Efficacy data not available. |
| SU 2015(26) | Case report | eGFR of 4 (serum creatinine of 11.16mg/dL) | Gout flare prophylaxis and treatment | 0.6mg PO once or twice daily (for months), and for gout flare, there was increased dose to 0.9mg PO once daily for 7 days |  |  |  | 1 | 1 | Efficacy data not available. |
| VAN DER VALDEN 2008(27) | Case report | eGFR of 17 (serum creatinine of 369µmol/l) | Gout flare prophylaxis | 0.5mg PO once daily (for at least 1 year) |  |  |  | 1 | 1 | Efficacy data not available. |
| WRIGHT 2017(28) | Audit | Patients with eGFR of <60 were included. | Gout flare treatment | 0.5mg PO twice daily (mentioned for 10 patients) | 128 | | | | 128 | Efficacy data not available. |
| YU 2018(29) | Audit | Patients with eGFR of <20 were excluded. | Gout flare prophylaxis. | Mean dose of 0.53±0.15mg/d | 9 | | |  | 9 | Pooled result: less total gout flare in the colchicine arm (compared to steroids arm), but no significant differences in duration of flares in both arms. |
| IL-1-INHIBITORS | | | | | | | | | | |
| SCHLESINGER 2011(30) | Phase 2 RCT – an extension of a phase 2 RCT by So et al, 2010  (multi-centre) | Study participants with eGFR of <30 were excluded | Gout flare treatment | Canakinumab in varying doses (10, 25, 50, 90 or 150mg) | 95 | | |  | 95 | Pooled results: Significant clinical improvement with canakinumab 150mg dose. |
| SCHLESINGER 2012  (β-RELIEVED & β-RELIEVED-II)(31) | Two phase 3 RCTs, followed by extension studies for both trials (multi-centre) | Study participants with eGFR <25 were excluded | β-RELIEVED:  Canakinumab 100mg single dose (gout flare treatment) | Canakinumab 100mg single dose vs triamcinolone (TA) |  | 51 | 37 |  | 88 | Pooled results: canakinumab (CAN) delayed the time to first new flare and reduced gout re-flare over the 12-week study by 62% vs triamcinolone (TA). The mean number of new flares/patient was lower in CAN arm (0.19 vs 0.51). Good CAN efficacy remained throughout the extended study, with delayed time to first new flare and reduced risk of gout re-flare by 56% vs TA. The mean number of new flares/patient was lower in CAN arm (0.40 vs 0.87). |
|  |  |  | β-RELIEVED II:  Canakinumab 100mg single dose (gout flare treatment) |  |  | 72 | 22 |  | 94 |  |
| SO 2007(32) | Pilot, open-labelled study (single-centre) | Study participants with eGFR of <30 were excluded. | Gout flare treatment | Anakinra 100mg/d for 3 days | 2 | 5 | 2 | 1 | 10 | Pooled efficacy outcome: rapid clinical response (within 24-48 hours) with 50% of patients reported 90-100% pain improvement. |
| SO 2010(33) | Phase 2 RCT (multi-centre) | Study participants with eGFR of <30 were excluded | Gout flare treatment | Canakinumab in varying doses (10, 25, 50, 90 or 150mg) | 95 | | |  | 95 | Pooled results: Significant clinical improvement with canakinumab 150mg dose. |
| SUNKUREDDI 2011(34) | Post-hoc analyses of the RCTs (β-RELIEVED & β-RELIEVED-II)  ACR abstract | CKD stage 2-5 | Gout flare treatment | Canakinumab (CAN) 100mg single dose vs triamcinolone (TA) |  | 188 | | | 188 | Pooled efficacy outcome: less gout flare compared to TA arm (25.5% vs 47.4% with OR 0.38). Mean VAS at baseline were 73.9mm (CAN) vs 73.8mm (TA) and 72 hours post dose VAS were 38.5mm (CAN) vs 49.9mm (TA). |
| SUNKUREDDI 2013(35) | Post-hoc analyses of the RCTs (β-RELIEVED & β-RELIEVED-II)  EULAR abstract | CKD stage ≥3 | Gout flare treatment | Canakinumab (CAN) 100mg single dose vs triamcinolone (TA) |  | 65 | | | 65 | Pooled efficacy outcome: over 24-week study period, a relative risk ratio of 54% for time to first new gout flare. Median time to first new flare was >24 weeks with CAN vs 19 weeks. CA arm showed significant reduction in the renal parameters – microalbumin (urine) (-81.2mg/l (CA) vs 45.1mg/l (TA)), albumin/creatinine ratio (urine) (-6.38mg/mmol (CA) vs 9.12mg/mmol(TA)) and serum creatinine (-6.1µmol/l (CA) vs -3.6µmol/l (TA)). |
| SUNKUREDDI 2014(36) | Post-hoc analysis of an RCT (multi-centre)  EULAR abstract | CKD stage ≥3 | Gout flare treatment | Canakinumab pre-filled syringe (CAN-PFS) vs 150mg canakinumab subcutaneous with lyophilised powder (CAN-LYO) vs triamcinolone (TA) |  |  | 24 (CAN-PFS) vs 28 (CAN-LYO) | | 24 (CAN-PFS) vs 28 (CAN-LYO) | Pooled efficacy outcome: pain intensity difference by -14.6mm in CAN-PFS vs TA from 72 hours post-dose until -16.1mm difference in CAN-PFS vs TA 7 days post-dose. The two CAN arms were comparable. Over 12 weeks, a single dose of CAN-PFS had relative RR of 90% for time to first new gout flare vs TA. |
| TERKELTAUB 2009(37) | Crossover trial (multi-centre) | Study participants with eGFR of <30 were excluded. | Gout flare treatment | Rilonacept 320mg once-off loading dose in the first 2 weeks, followed by 160mg weekly injection for week 3-7 | 2 | | |  | 2 | Pooled efficacy outcome: self-reported pain VAS significantly improved from 2-week placebo baseline to week 4, and with sustained improvement at week 8. 5 out of 10 patients reported 75% improvement. hsCRP level declined significantly. |
| TERKELTAUB 2012(38) | Post-hoc analyses of RCTs (PRE-SURGE 1, PRE-SURGE 2 and RE-SURGE)  ACR abstract | Study participants with eGFR of <20 were excluded. | Gout flare treatment | 160mg rilonacept (R160) weekly injection vs 80mg rilonacept (R80) vs placebo |  | 483 (R160) vs 141 (R80) | 82 (R160) vs 21 (R80) |  | 565 (R160) vs 162 (R80) | Pooled results: median VAS was significantly reduced in rilonacept arm and with sustained improvement. No effect on the number of affected joint but rilonacept overall seemed to be effective when number and severity of symptoms in all joints were considered. Median hsCRP levels reduced from 0.4mg/dL at week 2 to 0.1, 0.1 and 0.2mg/dL at weeks 4, 6 and 8 respectively. |
| CHEN 2010(39) | Case series (single-centre) | eGFR of <60 (serum creatinine range 1.1-3.0mg/dL) | Gout flare treatment | Anakinra – mean number of injections was 3.2 |  | 2 | 5 | 3 | 10 | Good response in 7 patients (duration of anakinra use in the range of 15-45 days); partial response in 3 patients (duration of anakinra use in the range of 4-30 days); 1 non-responder. |
| DONMEZ 2014(40) | Case report | eGFR of 55 (serum creatinine of 1.4mg/dL) | Gout flare treatment | Anakinra 100mg/d (for at least 4 months) |  |  | 1 |  | 1 | Good clinical response with ongoing treatment. |
| FUNCK-BRENTANO 2011(41) | Case report | ESRD on dialysis with eGFR of 8 | Gout flare treatment | Anakinra 100mg every other day (for at least 1 year) |  |  |  | 1 | 1 | Good clinical response with ongoing treatment. |
| GHOSH 2013(42) | Case series (single-centre) | Mean eGFR of 25 (range 18-41) | Gout flare treatment | Anakinra 100mg/d for those with normal renal function and stage 3 CKD and anakinra 100mg every 2-3 days for those with eGFR of <30 |  |  | 5 | | 5 | Pooled efficacy result: 67% of patients had pain improvement within 24 hours, with complete resolution achieved by day 5 in 72.5% of patients. |
| GRATTON 2009(43) | Case report | eGFR of 15 (serum creatinine of 4.3mg/dL) | Gout flare treatment | Anakinra 100mg (up to 4 times weekly) with concomitant prednisolone 7.5mg/d use |  |  |  | 1 | 1 | Good clinical response with ongoing treatment in addition to corticosteroids. |
| MCGONAGLE 2007(44) | Case report | CrCl of 54mL/min/1.73m^2^ | Gout flare treatment | Anakinra 100mg/d (for at least 6 months) |  |  | 1 |  | 1 | Good clinical response with ongoing treatment. |
| OTTAVIANI 2013(45) | Case series (multi-centre) | CKD stages 3-5 | Gout flare treatment | Anakinra - 23 patients had 100mg/d for 3 days, 7 patients on 100mg/d for <15days, and 10 patients on 100mg/d for >15days (range of duration, 2.3-11.8 months) |  |  | 40 | | 40 | 90% with good clinical response. Median pain VAS rapidly decreased from 73.5 to 25.0 and similarly with CRP level. |
| PALMA 2016(46) | Case series (single-centre)  ACR abstract | CKD stage 3 (mean eGFR of 49) | Gout flare treatment | Anakinra - 4 patients required a single dose of 100mg and 14 patients required 2-3 doses |  |  | 18 |  | 18 | Efficacy data not available. |
| SINGH 2009(47) | Case report | CKD stage 3 | Gout flare treatment | Anakinra 100mg/d (for at least 7 months) |  |  | 1 |  | 1 | Good clinical response with ongoing treatment. |
| NON-STEROIDAL ANTI-INFLAMMATORY DRUGS | | | | | | | | | | |
| MIKHNEVICH 2013(48) | Case series | Variable eGFR | Gout flare treatment | Diclofenac variable dosing (range 175-200mg) | 82 | | 15 | | 97 | Efficacy data not available. 53% of patients had decline in renal function. |
| GLUCOCORTICOIDS | | | | | | | | | | |
| SUNKUREDDI 2014(36) | Post-hoc analysis of an RCT (multi-centre)  ACR abstract | CKD stage ≥3 | Gout flare treatment | Triamcinolone 40mg IM |  |  | 24 | | 24 | Efficacy data not reported independently for triamcinolone. |
| BAJAJ 2004(49) | Case series (single-centre) | Variable eGFR | Gout flare treatment | Mean prednisone dose of 8mg/d | 4 | | 2 | 4 | 10 | Gout flare prevention was not achieved. |
| FARGETTI 2012(50) | Case report | eGFR of 38 (serum creatinine of 1.9mg/dL) | Gout flare treatment | Prednisone 5-20mg/d |  |  | 1 |  | 1 | Effective gout flare control after 43 days of treatment. |
| HAUSCH 1991(51) | Case report | eGFR of 50 (serum creatinine of 1.53mg/dL) | Gout flare treatment | Prednisone 10-20mg/d |  |  | 1 |  | 1 | Effective gout flare control after increase in prednisone dose. |
| HILL 2008(52) | Case report | eGFR of 15 | Gout flare prophylaxis | Prednisone 40mg/d |  |  |  | 1 | 1 | Ineffective for prevention of gout flare prior to rasburicase infusion. |
| KARIMZADEH 2009(53) | Case report | eGFR of 58 (serum creatinine of 1.3mg/dL) | Gout flare treatment | Prednisone 30mg/d |  |  | 1 |  | 1 | Rapid improvement in joint symptoms. |
| MAEKAWA 2014(54) | Case report | eGFR of 13.7 | Gout flare treatment | Prednisone 20mg/d |  |  |  | 1 | 1 | Rapid relief in joint pain. |
| RICHETTE 2006(55) | Case report | CrCl of 29mL/min/1.73m^2^ | Gout flare treatment | Methylprednisolone 100mg IV |  |  |  | 1 | 1 | Major improvement in joint inflammation. |
| SARMENTO 2009(56) | Case report | CrCl of 37mL/min/1.73m^2^ | Gout flare prophylaxis | Prednisone 5mg/d |  |  | 1 |  | 1 | Improvement in frequency of gout flare. |
| UDAYAKUMAR 2010(57) | Case report | eGFR of 38 (serum creatinine of 1.8mg/dL) | Gout flare treatment | Prednisone 40mg/d |  |  | 1 |  | 1 | Efficacy data not available. |

ACR: American College of Rheumatology; APEX: Allopurinol- and Placebo-Controlled, Efficacy Study of Febuxostat; CKD: chronic kidney disease; CONFIRMS: A Phase 3, Randomized, Multicenter, Double-Blind, Allopurinol-Controlled Study Assessing the Efficacy and Safety of Oral Febuxostat in Subjects With Gout; CrCl: creatinine clearance; CRP: C-reactive protein; eGFR: estimated glomerular filtration rate; ESRD: end-stage renal disease; EULAR: European League Against Rheumatism; FACT: Febuxostat Versus Allopurinol Control Trial in Subjects With Gout; GI: gastrointestinal; GOSPEL 2: subgroup analysis of GOSPEL (*goutte et observation des strat*égies de prise en charge en médecine ambulatoire*)* survey; hsCRP: highly sensitive C-reactive protein; IL-1; interleukin-1; IV: intravenous; PO: per os (by mouth); PRE-SURGE 1: Preventative Study Against Urate-Lowering Drug-Induced Gout Exacerbations 1; PRE-SURGE 2: Preventative Study Against Urate-Lowering Drug-Induced Gout Exacerbations; RCT: randomized controlled trial; RCT: randomized controlled trial; RE-SURGE: Review of Safety Using Rilonacept in Preventing Gout Exacerbations; VAS: visual analogue scale; β-RELIEVED & β-RELIEVED-II: two phase three randomized studies (response in acute flare and in prevention of episodes of re-flare in gout)

**Supplementary Table 3: Safety outcome reporting of gout flare prophylaxis and therapy use without renal function stratification**

| First Author (Year)  (Trial Name) | Adverse/Serious Adverse Events Reported by Renal Function | Notable Findings |
| --- | --- | --- |
| COLCHICINE | | |
| AHERN 1987(1) | No | Pooled safety outcome: diarrhoea and/or vomiting was reported in the colchicine arm at a median time of 24 hours (range 12-36 hours) or after a mean dose of 6.7mg. |
| BORSTAD 2004(2) | No | Pooled safety outcome: overall adverse events were comparable in both arms (43% colchicine vs 36% placebo), with diarrhoea being the most common adverse event in the colchicine arm (38% vs 4.5%). |
| WORTMANN 2010  (FACT, APEX, CONFIRMS)(3) | No | Pooled safety outcome: overall adverse events of 55.1%, with most frequently reported adverse event of URTI. Diarrhoea was reported 3 times much more frequent compared to naproxen (8.4% vs 2.7%) in both FACT and APEX, but no difference in CONFIRMS. In CONFIRMS, 2.8% reported headache and 7.7% had abnormal LFT. |
| PASCART 2016  (GOSPEL 2)(4) | No | In the context of the overall study cohort, when both age and renal impairment variables were accounted for in the analysis, 18.6% of patients received inappropriate colchicine dosing. Adverse events reported for the overall study cohort include diarrhoea (76.1%), abdominal discomfort (69.5%), dyspepsia (49.7%), nausea (40.4%), stomach-ache (38%), pyrosis (26.2%), constipation (10.5%) and vomiting (10%). |
| SOLAK 2014(5) | No | Pooled safety outcome: adverse events reported (total, n) include persistent diarrhoea (3), nausea (2), vomiting (2), myalgia (7), alopecia (7), neuropathic symptoms (8), sensation deficit (2), muscle weakness (2) and muscle tenderness (3). |
| HUNG 2005(6) | No | Pooled safety outcome: higher mortality rate of 10.2% in the concomitant arm (vs 3.6% in the sequential arm) and 11.39% with pancytopenia in the concomitant arm (higher risk for those with ALT ≥2 times and higher median dose of colchicine). Severe adverse events were reported in 12 patients (death was reported in 8 patients). |
| KWON 2017(7) | No | Twelve patients developed myopathy (median time of onset: 48 days). Risk of myopathy was not found to increase with colchicine and concomitant statin use, even after adjusting for confounders. |
| AKAR 2001(8) | No | Colchicine neuromyopathy. |
| ALTIPARMAK 2002(9) | No | Colchicine neuromyopathy. |
| BAKER 2004(10) | No | Colchicine neuromyopathy in the context of concomitant statin use. |
| BOOMERSHINE 2002(11) | No | Colchicine-induced rhabdomyolysis. |
| CHENG 2005(12) | No | Fatal febrile neutropenia and subsequent death, in the context of concomitant clarithromycin use for pneumonia. |
| CHOI 1999(13) | No | Colchicine neuromyopathy and diarrhoea. |
| DIXON 2001(14) | No | Diarrhpea and severe neutropaenia. |
| HSU 2002(15) | No | Colchicine neuromyopathy in the context of concomitant statin use. |
| KUNCL 1987(16) | No | Colchicine neuromyopathy. |
| MARCINIAK 2016(17) | No | Colchicine neuromyopathy. |
| MONTSENY 1996(18) | No | Colchicine neuromyopathy in 2 patients and the other 2 patients had fatal colchicine toxicity (diarrhoea, blood dyscrasia), which resulted in death. |
| MORRIS 2003(19) | No | Adverse event was not reported. |
| MULLINS 2011(20) | No | Colchicine toxicity was reported in detail for 4 patients, with 3 of them resulted in death (2 patients had concomitant amiodarone use and 1 patient had inappropriate colchicine dose for the renal impairment). |
| NASHEL 1982(21) | No | Adverse event was not reported. |
| ORTEL 1974(22) | No | Adverse event was not reported. |
| PETERSEL 2007(23) | No | One patient with eGFR of 55 developed diarrhoea with subsequent dehydration and hypotension. |
| RANA 1997(24) | No | Colchicine neuromyopathy in the context of concomitant cyclosporin use for heart transplants. |
| RUTKOVE 1996(25) | No | Colchicine neuromyopathy (3 patients in the context of cyclosporin use for renal transplants). |
| SU 2015(26) | No | Colchicine neuromyopathy in the context of concomitant fluconazole use. |
| VAN DER VALDEN 2008(27) | No | Colchicine neuromyopathy in the context of concomitant clarithromycin use for pneumonia. |
| WRIGHT 2017(28) | No | Adverse event was not reported. |
| YU 2018(29) | No | 7.2% reported diarrhoea. No serious adverse event reported. |
| IL-1 INHIBITORS | | |
| SCHLESINGER 2011(30) | No | Canakinumab (CAN) treatment: serious adverse event was not reported. The incidence of adverse events as similar for CAN arm (41.3%) and triamcinolone (TA) arm (42.1%), except 2 mild-moderate adverse events (infection-related). |
| SCHLESINGER 2012  (β-RELIEVED & β-RELIEVED-II)(31) | No | Canakinumab (CAN) treatment (pooled results): adverse events occurred in 66.2% in the CAN arm compared to 52.8% in the TA arm (overall, mild-moderate severity). Most common adverse event is infection-related (20.4% in CAN arm vs 12.2% in triamcinolone (TA0 arm), and serious infection occurred only in the CA arm by 1.8% (abscess, pneumonia, gastroenteritis). 2 patients experienced injection site reaction with CA. One case of lipoma was seen in patient receiving CAN.  In the extension studies, there were notable decline in the platelet, neutrophil and white cell counts, as well as changes in the lipid profiles and a modest increase in serum urate in the CAN arm, without any complication. |
| SO 2007(32) | No | Anakinra treatment: adverse event was not observed. |
| SO 2010(33) | No | Canakinumab treatment (pooled results): 4 serious adverse events were reported – appendicitis, bronchitis, carotid artery stenosis and cerebrovascular disorder (all were thought to be not related to the study drugs). Incidence of infection was low (<11% overall). |
| SUNKUREDDI 2011(34) | No | Canakinumab treatment (pooled results): adverse events were observed in 66.5% (canakinumab (CAN)) vs 52.6% (triamcinolone (TA)). Serious adverse events (15 patients in the CAN arm and 6 patients in the TA arm) – not considered as treatment-related. Incidence of infections was comparable (7.4% in the CAN arm vs 7.3% in the TA arm). |
| SUNKUREDDI 2013(35) | No | Canakinumab treatment (pooled results): adverse events were observed in 73.8% in the canakinumab (CAN) arm vs 53.1% in the triamcinolone (TA) arm, most commonly hypertension (5 patients in the CAN arm) and arthralgia (3 patients in the TA arm). Serious adverse events were observed in 7 patients in the CAN arm (pneumonia, angina pectoris, gastritis, hyperglycaemia, cerebrovascular accident and intracranial haemorrhage (death), chronic renal failure, increased prostate specific antigen, device dislocation) and none in the TA arm. |
| SUNKUREDDI 2014(36) | No | Canakinumab (CAN) treatment (pooled results): serious adverse events were reported in 2, 4 and 1 patients in CAN-PFS (CAN liquid formulation), CAN-LYO (CAN lyophilised powder) and triamcinolone (TA) arms respectively, with infection being the most common cause. No death was reported. Adverse events were reported in 12, 11 and 10 patients in CAN-PFS, CAN-LYO and TA arms respectively, with infection being the most common cause. |
| TERKELTAUB 2009(37) | No | Rilonacept treatment (pooled results): 1 patient withdrew from the study due to severe injection site reaction and skin induration, and both adverse events resolved without sequelae. Most adverse events were mild, mostly injection site reactions. No death or serious adverse events were observed in the study. |
| TERKELTAUB 2012(38) | No | Rilonacept treatment (pooled results): the incidence of treatment-emergent adverse events and serious adverse events in patients with eGFR <60ml/min, respectively, were similar between treatment groups (64.2% and 8.6% in placebo arm vs 67.6% and 6.0% in all rilonacept arms).  The incidence of treatment-emergent adverse events and serious adverse events in patients with eGFR ≥60ml/min, respectively, were also similar between treatment groups (59.0% and 3.3% in placebo arm vs 65.6% and 3.0% in all rilonacept arms). |
| CHEN 2010(39) | No | Anakinra treatment: 1 patient (the non-responder) with an injection site reaction 1 week after treatment. |
| DONMEZ 2014(40) | No | Anakinra treatment: adverse event was not reported. |
| FUNCK-BRENTANO 2011(41) | No | Anakinra treatment: adverse event was not reported. |
| GHOSH 2013(42) | No | Anakinra treatment: adverse event was not observed. |
| GRATTON 2009(43) | No | Anakinra treatment: adverse event was not reported. |
| MCGONAGLE 2007(44) | No | Anakinra treatment: adverse event was not reported. |
| OTTAVIANI 2013(45) | No | Anakinra treatment: 7 infectious events were observed with long-term use. |
| PALMA 2016(46) | No | Anakinra treatment: 1 patient with worsening encephalopathy in the context of underlying decompensated liver failure. |
| SINGH 2009(47) | No | Anakinra treatment: adverse event was not observed. |
| NON-STEROIDAL ANTI-INFLAMMATORY DRUGS (NSAIDs) | | |
| MIKHNEVICH 2013(48) | No | Infections and symptoms of chronic heart failure likely in the context of NSAIDs use. These symptoms were more frequent in patients with reduced eGFRs. |
| GLUCOCORTICOIDS | | |
| SUNKUREDDI 2014(36) | No | Pooled safety data: 2 cases of infections (8.3%) and 1 case of serious infection (4.2%). |
| BAJAJ 2004(49) | No | Nine out of 10 patients with worsening renal function prior to gout flare in the context of underlying systemic lupus erythematosus. |
| FARGETTI 2012(50) | No | Adverse event was not reported. |
| HAUSCH 1991(51) | No | Adverse event was not reported. |
| HILL 2008(52) | No | Steroid-induced myopathy. |
| KARIMZADEH 2009(53) | No | Adverse event was not reported. |
| MAEKAWA 2014(54) | No | Adverse event was not observed. |
| RICHETTE 2006(55) | No | Adverse event was not reported. |
| SARMENTO 2009(56) | No | Adverse event was not reported. |
| UDAYAKUMAR 2010(57) | No | Adverse event was not reported. |

ALT: alanine aminotransferase; APEX: Allopurinol- and Placebo-Controlled, Efficacy Study of Febuxostat; CKD: chronic kidney disease; CONFIRMS: A Phase 3, Randomized, Multicenter, Double-Blind, Allopurinol-Controlled Study Assessing the Efficacy and Safety of Oral Febuxostat in Subjects With Gout; eGFR: estimated glomerular filtration rate; FACT: Febuxostat Versus Allopurinol Control Trial in Subjects With Gout; GOSPEL 2: subgroup analysis of GOSPEL (*goutte et observation des strat*égies de prise en charge en médecine ambulatoire*)* survey; LFT: liver function test; URTI: upper respiratory tract infection; β-RELIEVED & β-RELIEVED-II: two phase three randomized studies (response in acute flare and in prevention of episodes of re-flare in gout)

**References**

1. Ahern MJ, Reid C, Gordon TP, McCredie M, Brooks PM, Jones M. Does colchicine work? The results of the first controlled study in acute gout. Aust N Z J Med. 1987;17(3):301-4.

2. Borstad GC, Bryant LR, Abel MP, Scroggie DA, Harris MD, Alloway JA. Colchicine for prophylaxis of acute flares when initiating allopurinol for chronic gouty arthritis. J Rheumatol. 2004;31(12):2429-32.

3. Wortmann RL, Macdonald PA, Hunt B, Jackson RL. Effect of prophylaxis on gout flares after the initiation of urate-lowering therapy: analysis of data from three phase III trials. Clin Ther. 2010;32(14):2386-97.

4. Pascart T, Lancrenon S, Lanz S, Delva C, Guggenbuhl P, Lambert C, et al. GOSPEL 2 - Colchicine for the treatment of gout flares in France - a GOSPEL survey subgroup analysis. Doses used in common practices regardless of renal impairment and age. Joint Bone Spine. 2016;83(6):687-93.

5. Solak Y, Atalay H, Biyik Z, Alibasic H, Gaipov A, Guney F, et al. Colchicine toxicity in end-stage renal disease patients: a case-control study. Am J Ther. 2014;21(6):e189-95.

6. Hung IF, Wu AK, Cheng VC, Tang BS, To KW, Yeung CK, et al. Fatal interaction between clarithromycin and colchicine in patients with renal insufficiency: a retrospective study. Clin Infect Dis. 2005;41(3):291-300.

7. Kwon OC, Hong S, Ghang B, Kim YG, Lee CK, Yoo B. Risk of Colchicine-Associated Myopathy in Gout: Influence of Concomitant Use of Statin. Am J Med. 2017;130(5):583-7.

8. Akar A, Bulent Tastan H, Erbil H, Arca E, Kurumlu Z, Gur AR. Efficacy and safety assessment of 0.5% and 1% colchicine cream in the treatment of actinic keratoses. J Dermatolog Treat. 2001;12(4):199-203.

9. Altiparmak MR, Pamuk ON, Pamuk GE, Hamuryudan V, Ataman R, Serdengecti K. Colchicine neuromyopathy: a report of six cases. Clin Exp Rheumatol. 2002;20(4 Suppl 26):S13-6.

10. Baker SK, Goodwin S, Sur M, Tarnopolsky MA. Cytoskeletal myotoxicity from simvastatin and colchicine. Muscle Nerve. 2004;30(6):799-802.

11. Boomershine KH. Colchicine-induced rhabdomyolysis. Ann Pharmacother. 2002;36(5):824-6.

12. Cheng VC, Ho PL, Yuen KY. Two probable cases of serious drug interaction between clarithromycin and colchicine. South Med J. 2005;98(8):811-3.

13. Choi SS, Chan KF, Ng HK, Mak WP. Colchicine-induced myopathy and neuropathy. Hong Kong Med J. 1999;5(2):204-7.

14. Dixon AJ, Wall GC. Probable colchicine-induced neutropenia not related to intentional overdose. Ann Pharmacother. 2001;35(2):192-5.

15. Hsu WC, Chen WH, Chang MT, Chiu HC. Colchicine-induced acute myopathy in a patient with concomitant use of simvastatin. Clin Neuropharmacol. 2002;25(5):266-8.

16. Kuncl RW, Duncan G, Watson D, Alderson K, Rogawski MA, Peper M. Colchicine myopathy and neuropathy. N Engl J Med. 1987;316(25):1562-8.

17. Marciniak C, Babu A, Ghannad L, Burnstine R, Keeshin S. Unusual Electromyographic Findings Associated With Colchicine Neuromyopathy: A Case Report. PM R. 2016;8(10):1016-9.

18. Montseny JJ, Meyrier A, Gherardi RK. Colchicine toxicity in patients with chronic renal failure. Nephrol Dial Transplant. 1996;11(10):2055-8.

19. Morris I, Varughese G, Mattingly P. Colchicine in acute gout. BMJ. 2003;327(7426):1275-6.

20. Mullins M, Cannarozzi AA, Bailey TC, Ranganathan P. Unrecognized fatalities related to colchicine in hospitalized patients. Clin Toxicol (Phila). 2011;49(7):648-52.

21. Nashel DJ, Chandra M. Acute gouty arthritis. Special management considerations in alcoholic patients. JAMA. 1982;247(1):58-9.

22. Ortel RW, Newcombe DS. Acute gouty arthritis and response to colchicine in the virtual absence of synovial-fluid leukocytes. N Engl J Med. 1974;290(24):1363-4.

23. Petersel D, Schlesinger N. Treatment of acute gout in hospitalized patients. J Rheumatol. 2007;34(7):1566-8.

24. Rana SS, Giuliani MJ, Oddis CV, Lacomis D. Acute onset of colchicine myoneuropathy in cardiac transplant recipients: case studies of three patients. Clin Neurol Neurosurg. 1997;99(4):266-70.

25. Rutkove SB, De Girolami U, Preston DC, Freeman R, Nardin RA, Gouras GK, et al. Myotonia in colchicine myoneuropathy. Muscle Nerve. 1996;19(7):870-5.

26. Su YC, Wu CC. Colchicine-Induced Acute Neuromyopathy in a Patient Using Concomitant Fluconazole: Case Report and Literature Review. Drug Saf Case Rep. 2015;2(1):16.

27. van der Velden W, Huussen J, Ter Laak H, de Sevaux R. Colchicine-induced neuromyopathy in a patient with chronic renal failure: the role of clarithromycin. Neth J Med. 2008;66(5):204-6.

28. Wright S, Chapman PT, Frampton C, O'Donnell JL, Raja R, Stamp LK. Management of Gout in a Hospital Setting: A Lost Opportunity. J Rheumatol. 2017;44(10):1493-8.

29. Yu J, Qiu Q, Liang L, Yang X, Xu H. Prophylaxis of acute flares when initiating febuxostat for chronic gouty arthritis in a real-world clinical setting. Mod Rheumatol. 2018;28(2):339-44.

30. Schlesinger N, De Meulemeester M, Pikhlak A, Yucel AE, Richard D, Murphy V, et al. Canakinumab relieves symptoms of acute flares and improves health-related quality of life in patients with difficult-to-treat Gouty Arthritis by suppressing inflammation: results of a randomized, dose-ranging study. Arthritis Res Ther. 2011;13(2):R53.

31. Schlesinger N, Alten RE, Bardin T, Schumacher HR, Bloch M, Gimona A, et al. Canakinumab for acute gouty arthritis in patients with limited treatment options: results from two randomised, multicentre, active-controlled, double-blind trials and their initial extensions. Ann Rheum Dis. 2012;71(11):1839-48.

32. So A, De Smedt T, Revaz S, Tschopp J. A pilot study of IL-1 inhibition by anakinra in acute gout. Arthritis Res Ther. 2007;9(2):R28.

33. So A, De Meulemeester M, Pikhlak A, Yucel AE, Richard D, Murphy V, et al. Canakinumab for the treatment of acute flares in difficult-to-treat gouty arthritis: Results of a multicenter, phase II, dose-ranging study. Arthritis Rheum. 2010;62(10):3064-76.

34. Sunkureddi P, Bardin T, Alten R, Schlesinger N, Bloch M, Kiechle T, et al. Effect of IL-1_ Inhibition with Canakinumab Compared to Triamcinolone

Acetonide on Pain Intensity and New Flares in Gouty Arthritis Patients with

Chronic Kidney Disease Stage 2–5;63(Suppl 10).

35. Sunkureddi P, Bardin T, Alten R, Schlesinger N, Bloch M, Kiechle T, et al. Efficacy and safety of canakinumab in gouty arthritis patients with chronic kidney disease stage ≥3. Annals of the Rheum Dis. 2013;71:447.

36. Sunkureddi P, Toth E, Brown J, Kivitz A, Stancati A, Richard D, et al. Efficacy and safety of canakinumab pre-filled syringe in acute gouty arthritis patients with chronic kidney disease stage ≥3. Arthritis & Rheumatology. 2014;73(Suppl 2):1083.

37. Terkeltaub R, Sundy JS, Schumacher HR, Murphy F, Bookbinder S, Biedermann S, et al. The interleukin 1 inhibitor rilonacept in treatment of chronic gouty arthritis: results of a placebo-controlled, monosequence crossover, non-randomised, single-blind pilot study. Ann Rheum Dis. 2009;68(10):1613-7.

38. Terkeltaub R, Evans RR, Weinstein SP, Wu R, Schumacher HR. Rilonacept for gout flare prophylaxis in patients with chronic kidney disease: Analysis of 3 clinical trials. Arthritis & Rheumatology. 2012;64(Suppl 10).

39. Chen K, Fields T, Mancuso CA, Bass AR, Vasanth L. Anakinra's efficacy is variable in refractory gout: report of ten cases. Semin Arthritis Rheum. 2010;40(3):210-4.

40. Donmez S, Pamuk ON. Chronic tophaceous gout. J Rheumatol. 2014;41(3):554-5.

41. Funck-Brentano T, Salliot C, Leboime A, Zafrani L, Servais A, Larousserie F, et al. First observation of the efficacy of IL-1ra to treat tophaceous gout of the lumbar spine. Rheumatology (Oxford). 2011;50(3):622-4.

42. Ghosh P, Cho M, Rawat G, Simkin PA, Gardner GC. Treatment of acute gouty arthritis in complex hospitalized patients with anakinra. Arthritis Care Res (Hoboken). 2013;65(8):1381-4.

43. Gratton SB, Scalapino KJ, Fye KH. Case of anakinra as a steroid-sparing agent for gout inflammation. Arthritis Rheum. 2009;61(9):1268-70.

44. McGonagle D, Tan AL, Shankaranarayana S, Madden J, Emery P, McDermott MF. Management of treatment resistant inflammation of acute on chronic tophaceous gout with anakinra. Ann Rheum Dis. 2007;66(12):1683-4.

45. Ottaviani S, Molto A, Ea HK, Neveu S, Gill G, Brunier L, et al. Efficacy of anakinra in gouty arthritis: a retrospective study of 40 cases. Arthritis Res Ther. 2013;15(5):R123.

46. Palma C, Topping T, Tabechian D. Anakinra is effective and well tolerated in medically complex patients including transplant recipients with gout. Arthritis & Rheumatology. 2016.

47. Singh D, Huston KK. IL-1 inhibition with anakinra in a patient with refractory gout. J Clin Rheumatol. 2009;15(7):366.

48. Mikhnevich E, Lemiasheuskaya S. Factors associated with refractoriness to NSAIDS in GOUTY arthritis. Annals of the Rheum Dis. 2013;71(Suppl 3):444.

49. Bajaj S, Fessler BJ, Alarcon GS. Systemic lupus erythematosus and gouty arthritis: an uncommon association. Rheumatology (Oxford). 2004;43(3):349-52.

50. Fargetti S, Goldenstein-Schainberg C, Silva Abreu A, Fuller R. Refractory gout attack. Case Rep Med. 2012;2012:657694.

51. Hausch R, Wilkerson M, Singh E, Reyes C, Harrington T. Tophaceous gout of the thoracic spine presenting as back pain and fever. J Clin Rheumatol. 1999;5:335-41.

52. Hill CL, Lu TY, Cervelli M, Mathew T. Failure of rasburicase therapy in recurrent acute gout with tophi. Int J Rheum Dis. 2008;11:315-7.

53. Karimzadeh H, Mohtasham N, Karimifar M, Salesi M, Bonakdar ZS. A case of ochronosis with gout and Monckeberg arteries. Rheumatol Int. 2009;29(12):1507-10.

54. Maekawa M, Tomida H, Aoki T, Hishida M, Morinaga T, Tamai H. Successful treatment of refractory gout using combined therapy consisting of febuxostat and allopurinol in a patient with chronic renal failure. Intern Med. 2014;53(6):609-12.

55. Richette P, Bardin T. Successful treatment with rasburicase of a tophaceous gout in a patient allergic to allopurinol. Nat Clin Pract Rheumatol. 2006;2(6):338-42; quiz 43.

56. Sarmento JF, Cavalcante VDA, Sarmento MTR, Braz ADS, Freire EAM. Chronic tophaceous gout mimicking rheumatoid arthritis. Revista Brasileira de Reumatologia. 2009;49:741-6.

57. Udayakumar D, Kteleh T, Alfata S, Bali T, Joseph A. Spinal gout mimicking paraspinal abscess: A case report. J Radiol Case Rep. 2010;4(6):15-20.
